# “Climatic fluctuations in the hyperarid core of the Atacama Desert during the past 215 ka”

**DOI:** 10.1038/s41598-019-41743-8

**Published:** 2019-03-27

**Authors:** Benedikt Ritter, Volker Wennrich, Alicia Medialdea, Dominik Brill, Georgina King, Sascha Schneiderwind, Karin Niemann, Emma Fernández-Galego, Julia Diederich, Christian Rolf, Roberto Bao, Martin Melles, Tibor J. Dunai

**Affiliations:** 1 Institute of Geology & Mineralogy, University of Cologne, Cologne, Germany; 2 Institute of Geography, University of Cologne, Cologne, Germany; 3 Institute of Earth Surface Dynamics, University of Lausanne, Lausanne, Switzerland; 4 Institute of Neotectonics and Natural Hazards, RWTH Aachen University, Aachen, Germany; 5 Leibniz Institute for Applied Geophysics (LIAG), Hannover, Germany; 6 Centro de Investigacións Científicas Avanzadas (CICA), Facultade de Ciencias, Universidade da Coruña, Coruña, Spain

## Abstract

Paleoclimate records from the Atacama Desert are rare and mostly discontinuous, mainly recording runoff from the Precordillera to the east, rather than local precipitation. Until now, paleoclimate records have not been reported from the hyperarid core of the Atacama Desert (<2 mm/yr). Here we report the results from multi-disciplinary investigation of a 6.2 m drill core retrieved from an endorheic basin within the Coastal Cordillera. The record spans the last 215 ka and indicates that the long-term hyperarid climate in the Central Atacama witnessed small but significant changes in precipitation since the penultimate interglacial. Somewhat ‘wetter’ climate with enhanced erosion and transport of material into the investigated basin, commenced during interglacial times (MIS 7, MIS 5), whereas during glacial times (MIS 6, MIS 4–1) sediment transport into the catchment was reduced or even absent. Pelagic diatom assemblages even suggest the existence of ephemeral lakes in the basin. The reconstructed wetter phases are asynchronous with wet phases in the Altiplano but synchronous with increased sea-surface temperatures off the coasts of Chile and Peru, i.e. resembling modern El Niño-like conditions.

## Introduction

**Figure 1:**
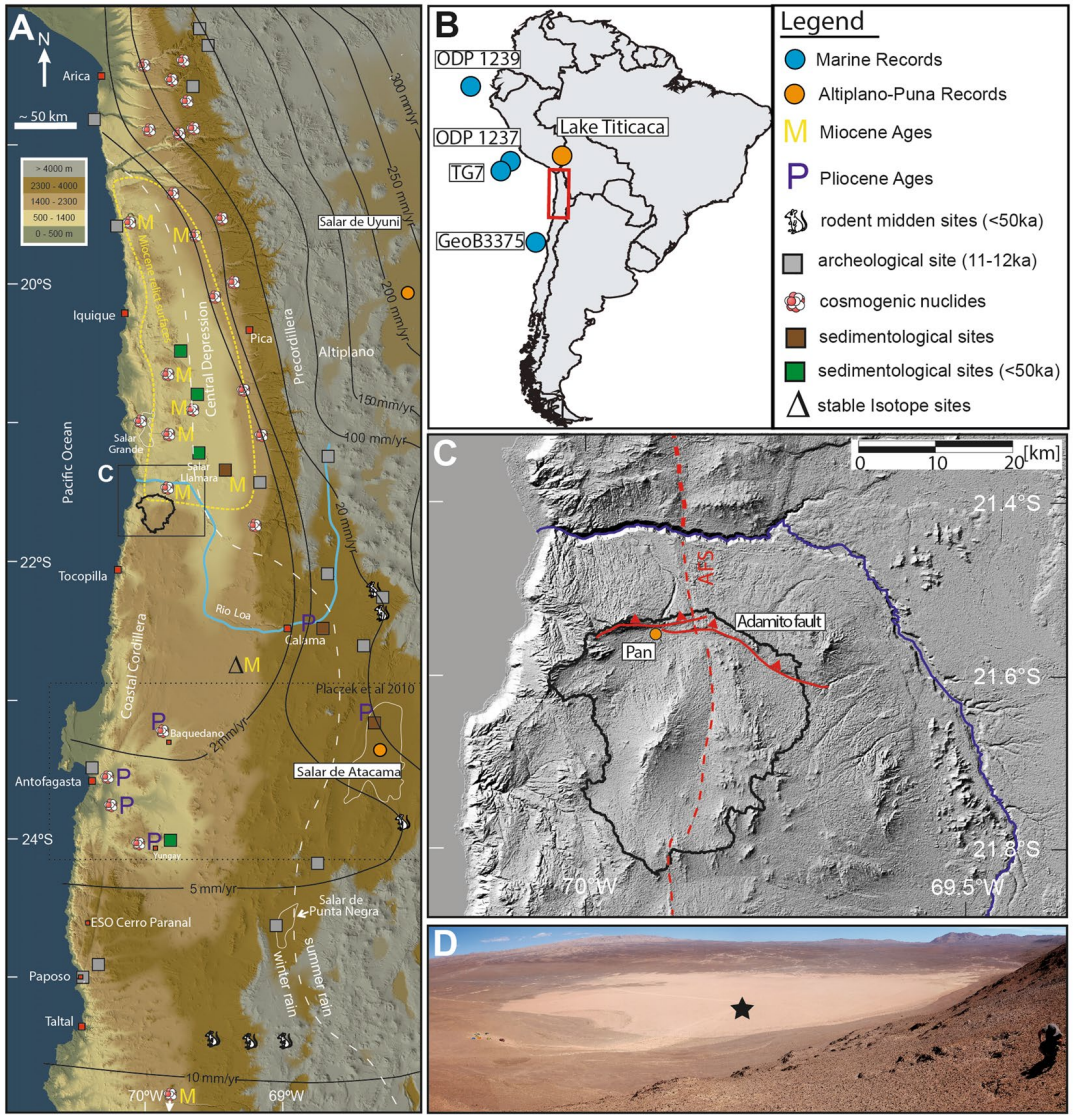
(**A**) Colour shaded digital elevation model (derived from SRTM-data, created using ArcGIS 10.5.1) with isohyets^1^ after Ritter, *et al*.^31^. Dashed white line indicates the border between winter-rain dominated areas in the SW and summer-rain dominated areas in the^1^. Sites from the literature are: rodent midden sites^23,64^, earliest archaeological sites^65^, stable isotope studies^66^, sedimentological studies^14,25,67,68^, cosmogenic nuclide exposure ages and erosion rates determined with cosmogenic nuclides^9–12,68–74^. The stippled yellow outline for Miocene relict surfaces is derived from studies yielding Miocene exposure ages (M) for sediment surfaces^11,12,70,71^ and sedimentological studies^67^. Studies yielding Pliocene ages for the onset of aridity are marked with P. The study area is marked with a black rectangle and drainage catchment of the sediment record is encircled with a black line. (**B**) Overview map of South America with positions of reference studies marked with coloured circles and the location of the Atacama Desert marked with a red rectangle. (**C**) Hillshade image based on Aster GDEM data (30 m resolution, produced using ArcGIS 10.5.1). Red lines indicate major tectonic fault systems (AFS = Atacama Fault System). The coring site (orange circle) is located in the mud pan in the northern part of an endorheic basin, whose drainage catchment is indicated with the black line. (**D**) View from the North onto the mud pan with the drilling location indicated by a star (Photo made by V. Wennrich).

The Atacama Desert of northern Chile is one of the driest places on Earth; its extreme hyperarid core receives less than 2 mm/yr of precipitation^1^. These hyperarid conditions are the consequence of subtropical atmospheric subsidence^2^ and temperature inversion due to coastal upwelling of the cold Peru-Chile Current (PCC) (or Humboldt Current^1^). Furthermore, a continental effect (distance to the Atlantic) and rainshadow effect at the eastern flank of the Andes (Fig. 1A) effectively harvests sparse moisture arriving from the Atlantic^3–6^. High evaporation rates in most areas of the Atacama Desert additionally intensify the hyperarid conditions^3,7^. Although the main factors causing aridity are known, the onset of predominantly hyperarid conditions and variability in the degree of aridity in this area remain disputed^8–18^. Presently the core of the hyperarid Atacama Desert covers parts of the Coastal Cordillera and Central Depression between 22–19°S (Fig. 1A). Water availability increases, albeit moderately, away from this core. The Mean Annual Precipitation (MAP) increases rapidly from less than 20 mm/yr at 2300 m to over 300 mm/yr at 5000 m elevation^3^. At the southern edge of the hyperarid core, moisture sources are connected to south-westerly airflows that allow extratropical cyclone systems to migrate northwards bringing moisture to areas south of 24°S during austral winter^1^. These northward migrating cut-off low systems can penetrate further north, as occurred during recent flood events such as in March 2015^19,20^. Similar rain events in the (sub-) recent past may be reflected by widespread traces of fluvial erosion and transport in the southernmost region of the dry core (i.e. south of 21°30′S), whereas comparable features are largely absent further to the north.

Regional climate records from the Coastal Cordillera and the Central Depression are scarce and where available mostly cover only the Holocene and the Last Glacial Maximum (LGM)^6,16,18,21–25^. Climate records spanning the mid to late Pleistocene, exist from lake sediment sequences in the Bolivian Altiplano^26^ and from near-shore marine sediment-cores^27,28^, all of which are >400 km away from the desert interior, and cross steep climatic gradients^1,3^ (Fig. 1B). In this paper, we present a first mid to late Pleistocene record of precipitation changes close to the driest part of the Atacama as preserved in lacustrine clay pan sediments of a tectonically blocked endorheic basin within the Coastal Cordillera.

## Site Information

The study site is situated in the Coastal Cordillera, which forms a 1000–1800 m high and 50–70 km wide structural high that is restricted to the west by a 1000 m coastal cliff and bounded to the east by the Central Depression (Fig. 1A,C). The studied clay pan (21° 32.5″S, 69° 54.8″W) is located at the northern end of an endorheic basin (Fig. 1C), which was formed by tectonic drainage displacement by reverse faulting (Adamito or Auguire fault)^29–31^. The clay pan at its terminus has a maximum surface area of 640 by 1000 m, and a catchment area of 560 km^2^ (Fig. 1A,C). Since the catchment is entirely contained within the Coastal Cordillera, and completely disconnected from groundwater influences from the Central Depression to the east, sediments in the terminal lake potentially record past fluvial processes from local to regional rainfall sources rather than from areas outside the Coastal Cordillera.

## Three-dimensional Basin Infill

**Figure 2:**
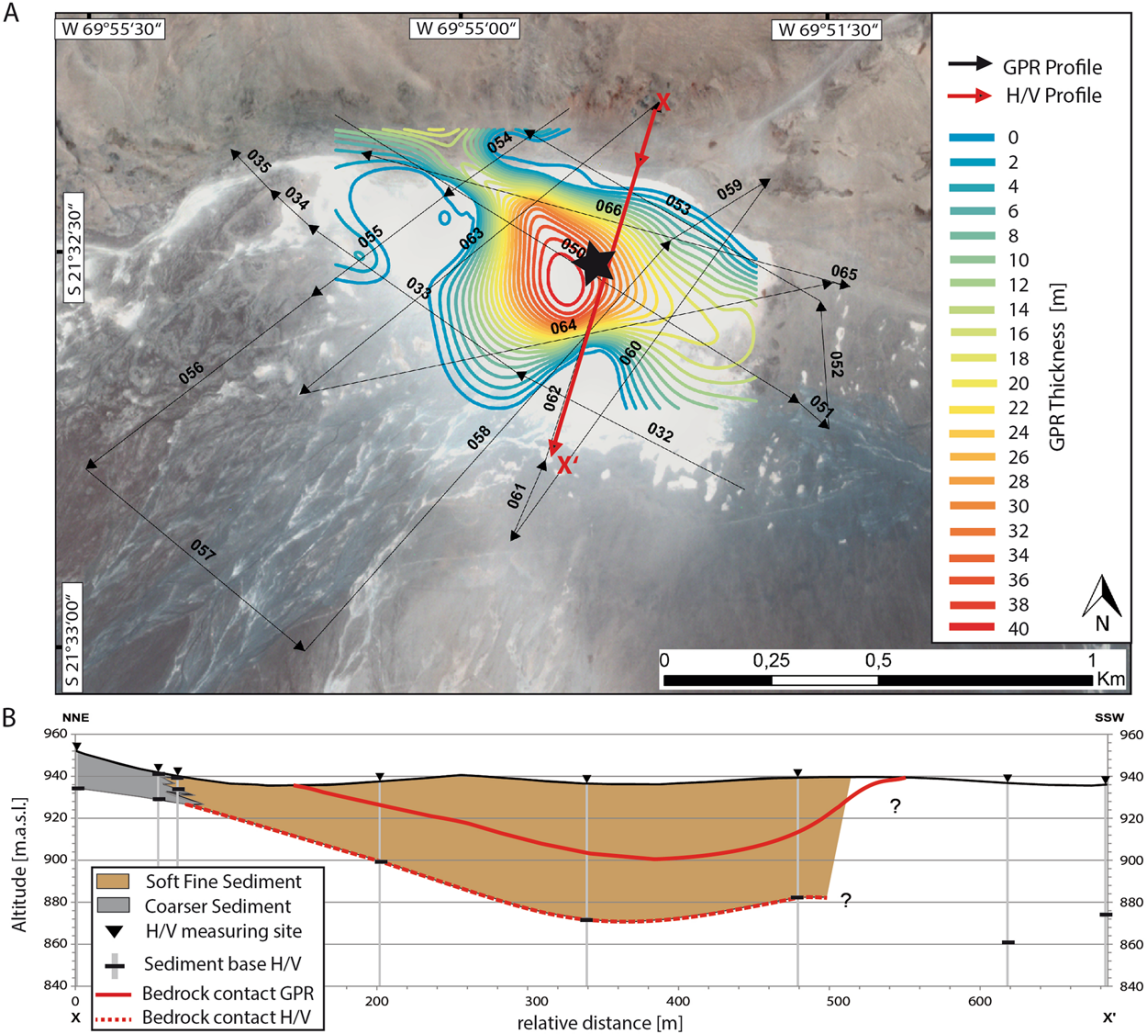
(**A**) Google Earth image (Image data: ©2018 CNES/Airbus & Digital Globe, image recoding 11/7/2014) including GPR and H/V profiles and GPR modelled contour lines of bedrock contact. Location of the drill site is indicated by a star. (**B**) Basin profile (solid red line in (**A**) derived from GPR (GPR data and extrapolation of bedrock contact based on inclination, solid red line) and H/V seismic (dashed red line). Bedrock contact derived from GPR (solid red line) is lower than that derived from H/V seismic derived (dashed red line).

The superficial extent of the clay pan is obvious from satellite imagery (Figs 1C and 2A). The three-dimensional basin morphology was reconstructed from dipping reflectors revealed from a net of Ground Penetrating Radar (GPR; for further information, see supplementary) profiles. As visible in Fig. 2B, the dipping appears to only be flat-angle throughout the basin, and can be extrapolated to a maximum depth of 41 ± 10 m at the centre of the clay pan (Fig. 2A,B). According to the GPR data, the basin morphology is not concentric and symmetric, but exhibits a shallower dipping at the southern part towards the centre and much steeper slopes at the thrusting scarp in the north (Fig. 2A,B). H/V seismic data (Fig. 2A profile red line, for further information see supplementary) were able to resolve soft sediment/bedrock contact, with a maximum depth of approximately 64 +/− 10 m (Fig. 2B). Due to the low soft-sediment density variations, a higher resolution model of the internal structure of the sediment infill could not be obtained from H/V seismic analysis.

## Lithological Characterisation

**Figure 3:**
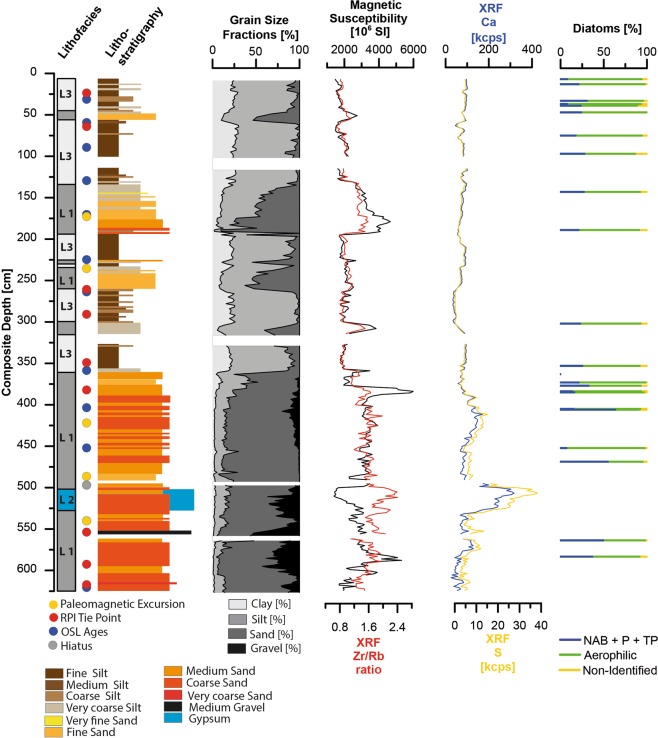
Lithofacies, lithology, grain-size distributions, magnetic susceptibility (MS), Zr/Rb ratio, S and Ca intensities, diatom assemblages and lithofacies of the record. Data gaps are due to recovery loss during drilling. Coloured dots mark age tie points, see age-depth model (Fig. 5C).

In 2014 and 2015 the uppermost 6.2 m of the sediment record (composite of PAG5 and 6) in the central part of the clay pan were recovered using hand-held percussion drilling system (Eijkelkamp). No macroscopically visible organic remains were found. Relative XRF intensities of sulphur (S) and calcium (Ca) are mostly stable throughout the sediment core, only between ~500–525 cm depth, S and Ca counts increase fourfold as compared to remainder of the core. The almost perfect correlation of S and Ca counts (R^2^ of 0.98) indicate the presence of calcium sulphate as sulphur bearing phase, in the interval between ~500–525 cm gypsum is macroscopically visible. We observe a strong positive correlation between the sediment grain-size and the Zr/Rb ratio (Fig. 3). Low Zr/Rb ratios are found in the silt to clay fraction, Zr is enriched in the sand fraction. Magnetic susceptibility covaries with increasing grain size and indicates a grain-size dependency. The core can be subdivided into three major sediment types (Lithofacies), differing distinctly in grain size, evaporite content, and inorganic geochemistry.

A coarser sediment type (Lithofacies 1), consisting predominantly of fine to medium sand and gravel, dominates the sediment record from 360 to 625 cm depth (Fig. 3). The abundance of gravel, especially medium gravel (>6.3 mm) increases towards the base of the core, with up to 50 wt.% for in few samples. The grain-size distributions (GSD) < 2 mm are unimodal. Above 360 cm Lithofacies 1 occurs as intercalation within Lithofacies 3, with bi- to trimodal GSDs (Fig. 3, see supplementary). Magnetic susceptibility exhibits high values, coincident with coarse grain-sizes. Lithofacies 2 is found in a brief interval between ~5.00–5.25 m and is a matrix supported gypsum-cemented sandy gravel (Fig. S3, see Supplementary Fig. S3), resembling a gypsum duricrust. This unit has the lowest magnetic susceptibility in the core, which can be attributed to a dilution effect due to the high gypsum content. Lithofacies 3, fine- to medium silt-size sediments with polymodal GSDs, constitutes the uppermost part of the section (0–360 cm). Intercalations of Lithofacies 1 occasionally interrupt Lithofacies 3 (Fig. 3). In line with the fine grain-size magnetic susceptibility is low.

## Biostratigraphical Characterisation

**Figure 4:**
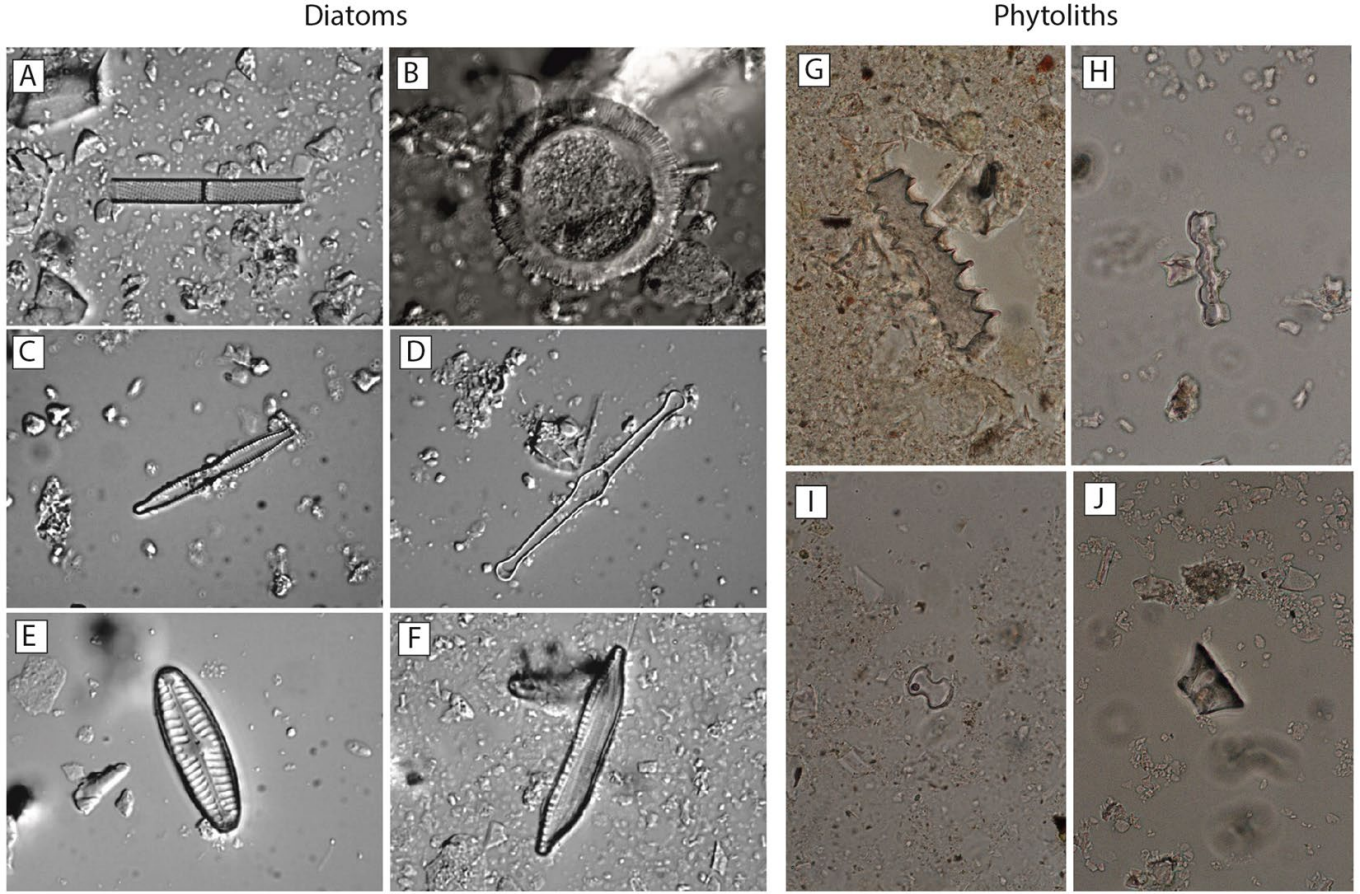
(**A**–**F**) Photographs of some representative diatom taxa (1000x magnification). (**A**) *Aulacoseira* cf. *ambigua* (Grunow) Simsonsen (**B**) *Cyclotella* sp. (**C**) *Fragilaria mesolepta* Rabenhorst (**D**) *Tabellaria flocculosa* (Roth) Kützing (**E**) *Pinnularia* cf. *borealis* (**F**) *Hantzschia amphioxys* (Ehrenberg) Grunow. (**G**–**J**) Photographs of some phytoliths found in the samples (1000x magnification).

Phytoliths were found in every sample, however, they were not further analysed for this study. Diatom frustules were found in 22 of the 28 samples examined (Figs 3 and 4, see Supplementary Dataset Fig. S5). They were absent in the 5 samples from the interval 616 to 505 cm and at 363 cm depth. The diatom-free horizons could be the result of either taphonomic processes preventing the preservation of diatoms, or of prevailing dry conditions incompatible with the presence of diatoms. Where present, diatoms in general are scarce and highly fragmented. Most samples are characterized by highest percentages of aerophilic species, primarily *Pinnularia borealis* Ehrenberg*, Hantzschia amphioxys (*Ehrenberg) Grunow and *Denticula* cf. *elegans* Kützing. An allochthonous transport of non-aerophilic diatom taxa into the clay pan cannot be excluded, but is rather unlikely due to the lack of lacustrine deposits within the drainage catchment. Nevertheless, non-aerophilic benthic and, in some cases, tychoplanktonic and euplanktonic diatoms were also found in low abundances, indicating the presence of shallow, ephemeral water bodies in the clay pan during the time of deposition (Fig. 4). This pattern is interrupted at 406 cm (Fig. 4), where the percentage of aerophilic diatoms drops below 30%, and non-aerophilic benthic (e.g., *Cocconeis placentula* Ehrenberg, *Tabularia fasciculata* (C. Agardh) D.M. Williams & Round), tychoplanktonic (e.g. *Fragilaria* spp.) and even euplanktonic species (mainly *Aulacoseira* spp.) reach their maximum abundances in the sequence. This sharp change in the diatom assemblage is compatible with deeper and more permanent lacustrine conditions in the clay pan. In the samples above 406 cm the diatom assemblage is again dominated by aerophilic taxa, illustrating the recurrence of the occasional presence of only shallow ephemeral water bodies. Aerophilic diatoms can inhabit a range of different subaerial or terrestrial habitats^32^. Among them, *Pinnularia borealis*, the main component of diatom assemblages in the upper part of the sediment sequence, seem to prefer the driest habitats^33^. It is one of the main freshwater algae taxa found in soil crusts of hot deserts around the world, i.e.^34^, and its high relative abundances seems compatible with the existence of arid to hyper-arid conditions. We cannot differentiate between the allochthonous input from the catchment and the autochthonous presence of aerophilic diatoms. Detailed information about specific taxa distributions can be found in the Supplementary Dataset.

## Core Chronology

### Optically Stimulated Luminescence (OSL)

The age-depth modelling followed a multi-faceted approach using OSL ages together with paleomagnetic tie points. OSL ages are based on the measurement of polymineralic fine-grained (4–11 µm) extracts of each sample, and were measured using post-infrared infrared stimulated luminescence at 225 °C (pIR-IR225^35^). The suitability of this protocol was determined through a series of dose recovery experiments. A fraction of each sample was bleached in a solar simulator and then either measured, to quantify the residual dose, or given a beta dose and then measured to calculate the recovered to given dose ratio. Samples show residual doses of between 1.9 and 6.7 Gy, which account for less than 3% of the respective burial doses across both signals. The recovered to given dose ratios are consistent with unity within 1σ for all samples. Both tests confirm the suitability of the protocol used.

**Figure 5:**
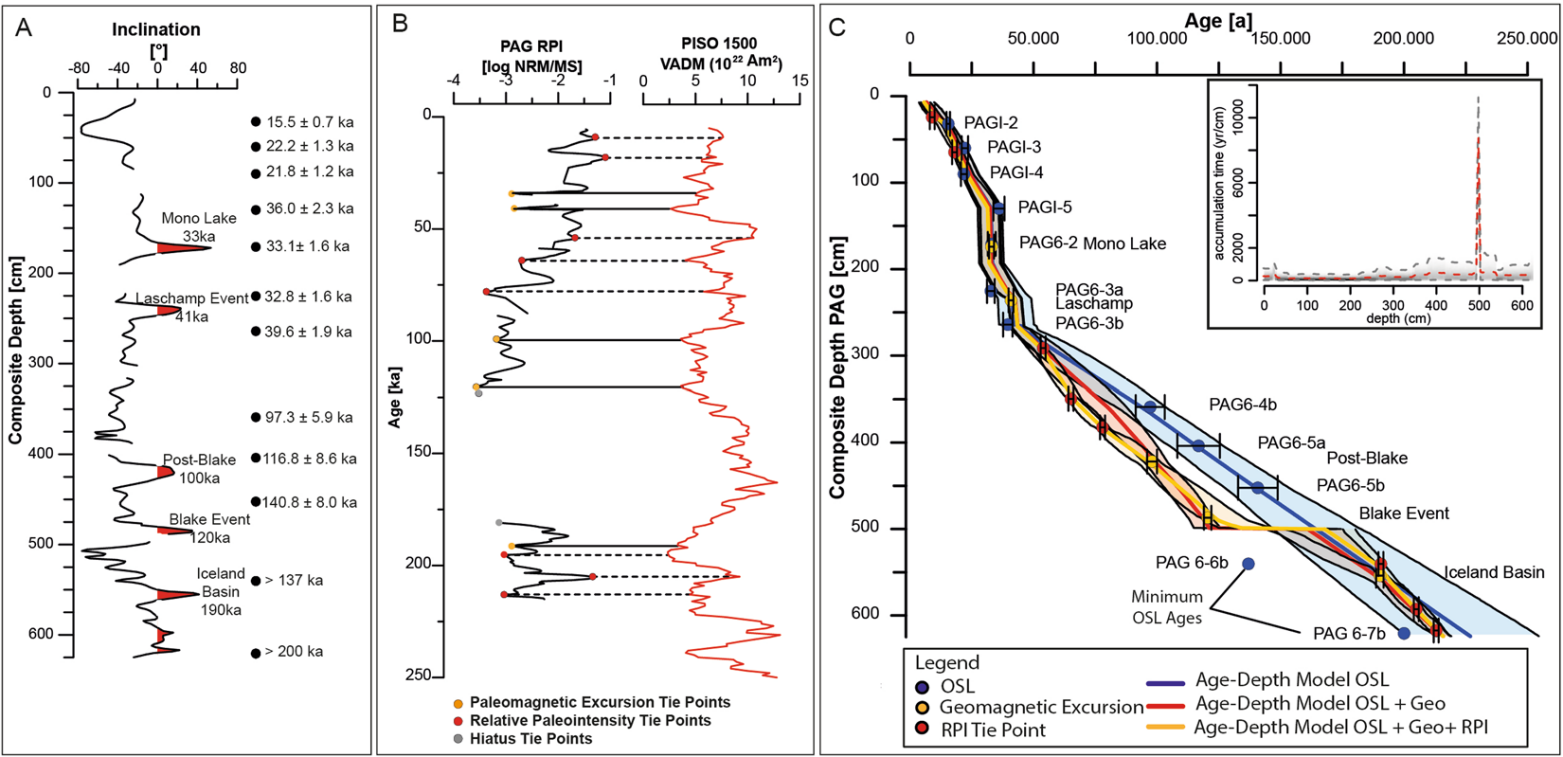
(**A**) Paleomagnetic inclination record of core PAG 6 from the PAG mud pan. Geomagnetic inclination excursion recorded in the sediment record are marked in red with proposed geomagnetic events. Black dots display measured OSL ages. (**B**) Relative paleointensity (RPI) record vs. used RPI stacks PISO1500^40^. Orange dots and solid lines indicate geomagnetic excursion tie points, red dots and dashed lines mark RPI tie points. Grey dots mark tie points for resembling phases of sedimentation stagnancy (hiatus). (**C**) Bayesian age-depth model using ‘Bacon’ from Blaauw and Christen^48^. Orange dots indicate geomagnetic excursion tie points, red dots RPI tie points and blue OSL ages. Accumulation time is given in yr/cm (calculated using ‘Bacon’^48^ without event-layers). Note that abrupt changes in the sedimentation rate is tightly linked to used tie points and most likely does not reflect natural smooth transitions. The lowest OSL ages (PAG 6-6b, PAG 6-7b) represent minimum ages (see text).

All samples were screened for anomalous fading^36^. Fading measurements show an average fading rate (i.e. g-value) of <1.0%/decade for samples down to 500 cm depth, suggesting that fading correction is not required for these samples^35^. In contrast, the average g-value for the pIRIR_225_ signal of the two samples below 500 cm, PAG6-6b and PAG6-7b, is 4.8%/decade and these samples have been fading corrected using the model of Huntley^37^ and the approach of Kars, *et al*.^38^. The results show that the natural signal is above saturation of the corrected dose response curve (DRC), and thus only minimum ages based on 2D_0_ of the DRC can be estimated for these samples from deepest within the sediment core, Table 1. Final dose rates calculated and derived ages are summarized in Table 1. Measured radionuclide concentrations can be found in the Supplementary Dataset Table S1. The OSL ages are in stratigraphic order and are in agreement with the trends of the paleomagnetic chronology (Table 1, Fig. 5A), within 2σ uncertainties. The two stratigraphically lowest ages (PAG6-6b and PAG6-7b) are younger than the paleomagnetic tie points (Fig. 5C), which is due to the fact that these samples are in field-saturation.

**Table 1:** Calculated dose rates and derived ages.

Sample	Depth (m)	Environmental dose rate (Gy/ka)	Burial dose (Gy)	Age (ka)
PAG I-2	0.32	5.26 ± 0.23	81.6 ± 0.9	15.5 ± 0.7
PAG I-3	0.6	5.17 ± 0.23	115.0 ± 4.4	22.2 ± 1.3
PAG I-4	0.9	5.27 ± 0.23	115.0 ± 4.1	21.8 ± 1.2
PAG I-5	1.3	5.22 ± 0.24	188.0 ± 8.1	36.0 ± 2.3
PAG 6-2	1.7	4.38 ± 0.20	145.0 ± 3.0	33.1 ± 1.6
PAG 6-3a	2.25	4.85 ± 0.21	159.0 ± 2.8	32.8 ± 1.6
PAG 6-3b	2.64	4.85 ± 0.21	192.0 ± 3.5	39.6 ± 1.9
PAG 6-4b	3.59	4.47 ± 0.24	435.0 ± 13.0	97.3 ± 5.9
PAG 6-5a	4.03	3.89 ± 0.20	454.0 ± 24.0	116.8 ± 8.6
PAG 6-5b	4.52	3.50 ± 0.17	493.0 ± 14.0	140.8 ± 8.0
PAG 6-6b	5.40	3.90 ± 0.17	>534	>137
PAG 6-7b	6.20	3.55 ± 0.15	>712	>200

### Paleomagnetic data

Geomagnetic field excursions are short-term, usually a few thousands of years, periods of anomalous geomagnetic directional behavior that emerge from the mean ‘background’ normal secular variability of the Earth’s magnetic field^39^. For the last normal magnetic Brunhes chron, up to 17 excursions are recorded^39^. Geomagnetic turn-overs are attended by relative paleointensity (RPI) minima^40^. This fact makes geomagnetic excursions a valuable tool to date sediment archives beyond the temporal range of ^14^C and OSL dating. Characteristic directions (inclination) and amplitude changes of RPI proxy were used to identify age tie points to further constrain a reliable age-depth model (Fig. 5A,B). Global records indicate the occurrence of at least six geomagnetic excursions in the time interval between 15 and 250 ka, the relative age range of our record according to the OSL data (see Supplementary Dataset Table S2).

We used a multi-stage approach to identify geomagnetic excursion, taking into account the previously described OSL data, lithostratigraphy, and comparison of the measured RPI proxy to global RPI stacks^40,41^. Comparison of excursions and the RPI record from the clay pan to the global RPI stack PISO1500^40^ and to the record of known excursions (e.g.^42^) were used to generate age-depth tie points (Fig. 5A,B). The comparison of both RPI records (this record vs. PISO1500) indicates that the occurrence of geomagnetic excursions mostly coincides with RPI lows, as recorded in other archives^40^ (Fig. 5B).

Due to the complexity of the depositional environment, we have to assume phases of non-deposition or even erosion. The short durations of geomagnetic excursions have important consequences for the precision of sedimentary paleomagnetic recording, if the signal is acquired during low sedimentation rates. It implies that the recovered sediments may not have recorded all geomagnetic excursions and that RPI data will have gaps. Some geomagnetic excursions are reliably recorded, while others may not be recorded at all due to low sedimentation rates or even hiata^43^.

We find five distinct anomalous inclination excursions in our record (Fig. 5A). These features are interpreted as coeval to global time markers such as the Mono Lake excursion (Depth: 171 cm, 33 ± 1 ka)^39,44^, the Laschamp Event (Depth: 243 cm, 41 ± 1.5 ka^45^), the Post-Blake (Depth: 422 cm, 98 ± 2 ka^40^) and Blake Events (Depth: 486 cm 120.5 ± 1.5 ka^39,40^) and the Iceland-Basin excursion (Depth: 555 cm, 190.2 ± 1.77 ka^46^). An alternative assignment of one of the geomagnetic excursion, i.e. Blake excursion at 422 cm and subsequent shift of geomagnetic excursions, would indicate the occurrence of the Pringle Falls excursion (211 ± 1.5 ka^42^) at the lowest inclination excursion at 555 cm (Fig. 5A). This alternative assignment, however, results in an age-model that is in disagreement with the sedimentological findings (hiatus at the pedogenic gypsum horizon), we therefore do not consider it further (it is discussed in the supplementary data set). Note that application of the alternative assignment/age-model would not affect the main conclusions of this study (asynchonicity of wet phases in the Altiplano and the Coastal Cordillera; see below).

The identification and depth of Mono Lake is supported by an OSL (post-IR225) age at 33.1 +/− 1.6 ka (PAG 6-2). The Laschamp Event could be identified with very high confidence due to the proximal occurrence following the Mono Lake excursion and characteristic RPI minima at 41 ka (PISO1500 and this record). The assignment of the inclination excursion to the Laschamp Event implies that the existing OSL ages in this part of the record are slightly too young (PAG 6-3a, PAG 6-3b). Inclination excursions at 422 cm and 486 cm can be attributed to the Post-Blake (98 +/− 2 ka) and the Blake Event (120.5 +/− 1.7 ka). Both excursions are marked by a characteristic RPI pattern of two minima in PISO1500 stack and our RPI record (~420–490 cm). This assignment to those discrete geomagnetic excursions leads to an age offset in OSL ages of up to 30 ka (Fig. 5C), implying potential incomplete pre-bleaching of the sediment during transport into the clay pan and insufficient resetting of the OSL signal. As the two OSL ages adjacent to the geomagnetic excursion at ~555 cm represent only minimum ages, we assign this excursion to the next known geomagnetic event, the Iceland Basin (190 ± 5 ka).

Comparing our record to PISO1500^40^ using the Iceland Basin excursion, our RPI record fits the characteristic pattern of RPI during this time range (Fig. 5A,B). The comparison with the PISO-1500 RPI stack^40^ yields partial agreement for the last 215 ka (Fig. 5B). This provides additional and independent support for the core chronology. We identified five RPI minima associated to geomagnetic excursions and additionally eight tie points related to characteristic RPI trends (Fig. 5B). A preliminary age-depth model was calculated using AnalySeries 2.0^47^ and linear interpolation between assigned chronological tie points and calculated ages. With the data, age-depth sedimentation histories of the sediment record were modelled using the ‘rBacon’ (V2.3.3) Bayesian statistics approach of Blaauw and Christen^48^ (Fig. 5C). OSL data and geomagnetic excursion tie points indicate two potential event layer and/or episodes of high sedimentation at around ~33 ka (PAG6-2, PAG6-3a, Mono Lake) and ~40 ka (Laschamp, PAG6-3b) related to coarser layers between 131–191 cm and 236–264 cm (Fig. 5C). The OSL age at 130 cm (PAGI-5 36 ± 1.6 ka) is slightly too old and is possibly influenced by partial bleaching.

OSL data and geomagnetic excursion tie points indicate an episode of low or lacking sedimentation between ~135–168 ka (depth ~500 cm). This episode is characterized in the global PISO1500 RPI stack by a broad RPI maxima, which could not be found in our RPI record (Fig. 5A,B). This discrepancy in the correlation/assignment of our record with the PISO stack, together with sedimentological evidence (‘*costra*’ gypsum crust as indication of surface activity stagnancy, Fig. 3) indicates the existence of a hiatus in our sediment record (including gypsum crust ~500–525 cm, ~135–180 ka). Additionally, we identified a smaller hiatus of a few thousand years at the top of the sediment record. The occurrence of other smaller hiata is possible, but cannot be resolved.

## Paleoclimate and Sedimentation in the hyperarid Coastal Cordillera

**Figure 6:**
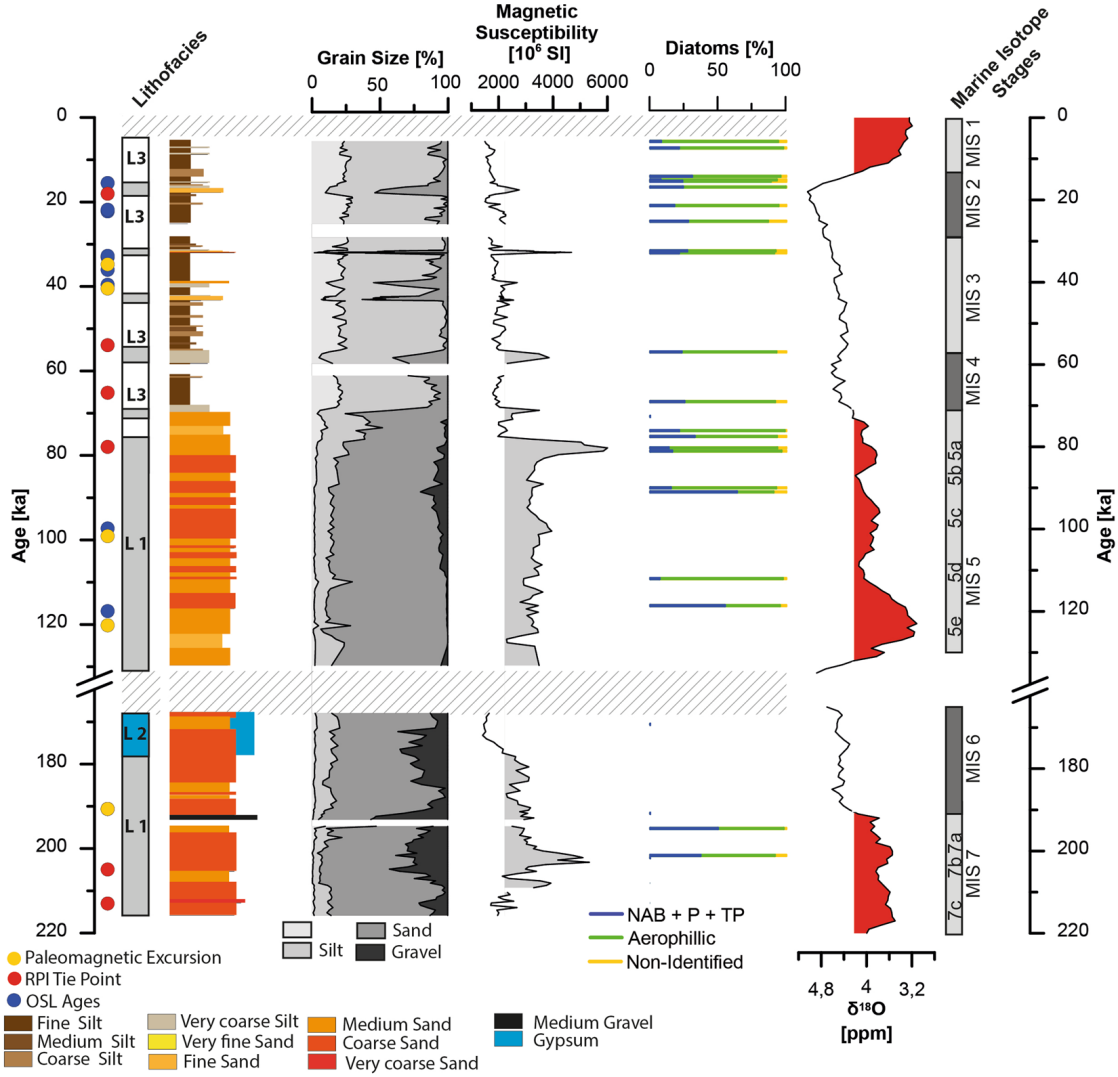
Lithofacies (L1, L2, L3), sediment types, grain size fractions (0–>6.3 mm), magnetic susceptibility (MS), and diatoms assemblages (non-aerophilic benthic (NAB), euplanktonic (P), tychoplanktonic (TP) in core PAG5/6 from the mud pan in the Atacama Desert, displayed against depth (for age-depth model see supplementary online material, coloured points mark age tie points) and the global deep-sea oxygen isotope stack with marine isotope stages from Lisiecki and Raymo^75^.

According to the chronology of core PAG 5,6 the investigated sediment succession covers the transition of MIS 7 to MIS 6 until the early Holocene (Fig. 6). However, the sediment sequence is not continuous. Besides presumed multi-year sedimentation breaks between rain events, two major hiata occur, from early MIS 6 to the onset of MIS 5, and from the mid Holocene to present. Hiata in the record may represent either erosion of fluvial sediments in the terminal pan and/or a paucity of sediment transported into the pan.

The sediments covering the end of MIS 7 until MIS 6, are dominated by coarse sand, e.g. high fluvial energy reflecting an allochthonous signal (Lithofacies 1; Fig. 6). The diatom assemblages found in these sediments are mostly composed of euplanktonic, tychoplanktonic and non-aerophilic benthic taxa, thus indicating water depths of a few metres at the time of deposition. Their occurrence in coarse-grained sediments points to high-energy discharge events of the main tributaries of the ephemeral lake, that created lacustrine environments favourable for non-aerophilic diatoms. A pedogenic gypsum crust in the uppermost ~25–30 cm of these coarse sediments (Lithofacies 2) marks a ~50 ka hiatus during the remainder of MIS 6.

Sedimentation resumed during the last interglacial (MIS 5e). Sand-sized sediments (Lithofacies 1) indicate high-energetic supply to the pan. However, the energy-level presumably was lower than during late MIS 7 and early MIS 6, as suggested by significantly lower concentrations of the coarse sand and gravel fractions (Fig. 6). The diatom assemblages in the MIS 5e deposits are variable. Euplanktonic, tychoplanktonic and non-aerophilic benthic diatoms characteristic of permanent/more evolved lacustrine water conditions, have total relative abundances > 50% during MIS 5e and MIS 5b. By contrast, aerophilic diatoms, typical of very shallow ephermal ponds, wet soils, or nearshore wetlands subjected to desiccation, dominate the assemblages during MIS 5d and MIS 5a-b.

Towards MIS 4 and throughout the Holocene, the sands are progressively replaced by finer, silt-sized deposits (Lithofacies 3), which along with the overall dominance of aerophilic diatoms indicate a period of reduced fluvial activity with temporary lacustrine conditions in shallow ponds (Fig. 6). Interspersed coarser sediments (Lithofacies 1) at ~33 and 40 ka, may indicate intense flooding events of the pan. The latter scenario could have its present analogue in the occurrence of lacustrine conditions after a major rain event in the Atacama Desert during the El Niño in 1997/98 creating a temporary lake in the Sechura Desert of southern Peru^49^, or in the occurrence of small ponds and lagoons after anomalous rain events in 2017 in the Yungay area of the Atacama Desert^50^.

**Figure 7:**
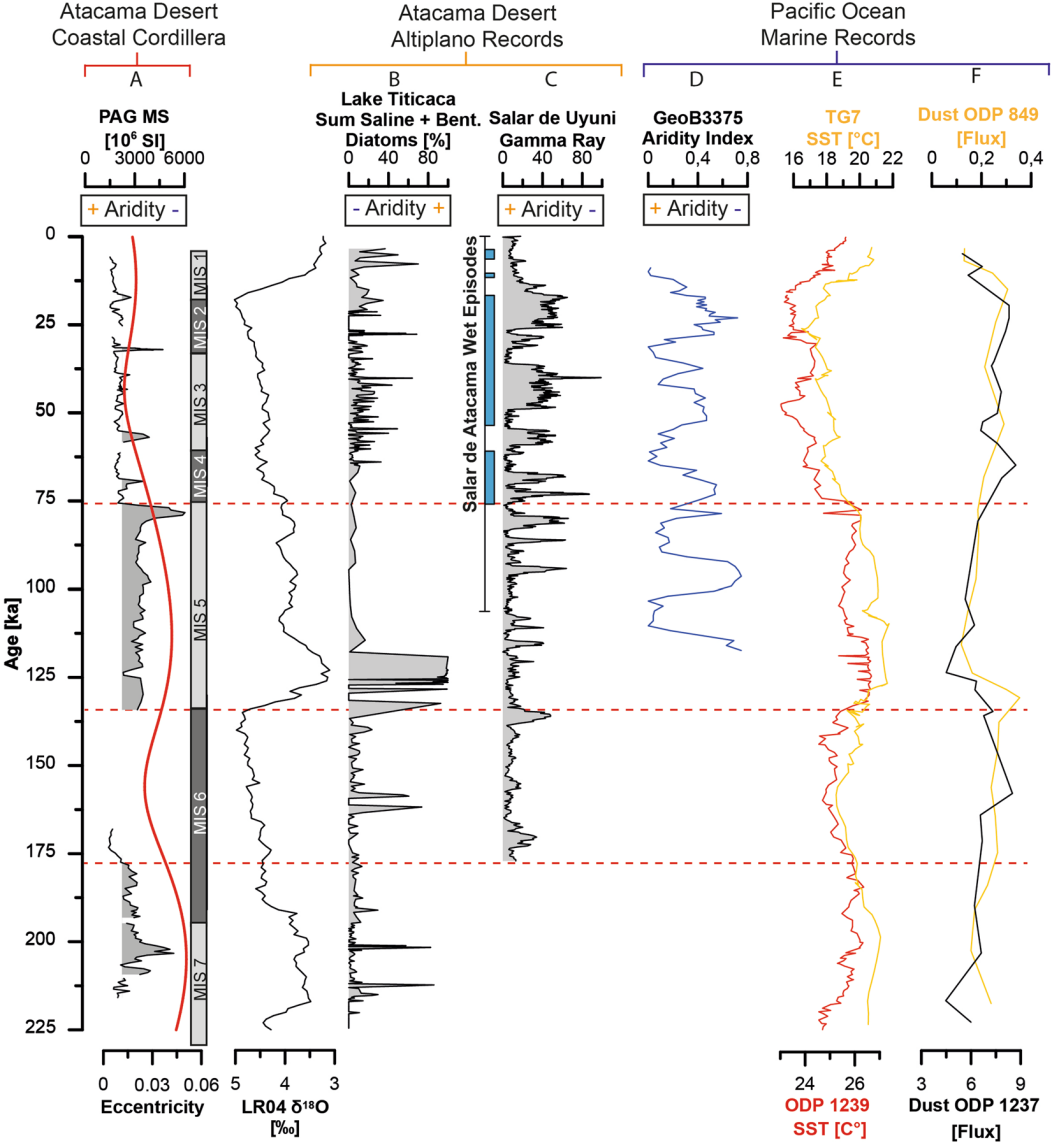
Paleoclimate record of fluvial episodes of the terminal pan record compared to selected regional and over regional terrestrial and marine paleo-records. (**A**) Magnetic susceptibility record and local eccentricity red line^76^. (**B**) Global deep-sea oxygen isotope stack^75^. (**C**) Lake Titicaca summation plot of saline and benthic diatoms species^26^. (**D**) Salar de Uyuni gamma-ray record^77^ and wet episodes from Salar de Atacama^54^. (**E**) Aridity index (27°S) reconstructed by Stuut and Lamy^57^. (**F**) SST reconstructions from the East Pacific at 17°S TG7^61^, and equatorial Pacific ODP 1239^59^. (**G**) Dust flux records from 16°S^60^ and the equatorial Pacific^62^.

In general, the dominance of benthic and aerophilic species with meso-polysaline to eurytopic salinity optima suggest episodes with shallow ephemeral ponds where elevated evaporation rates prompted higher salinity conditions. Those ponds could have originated from less frequent and small rain events. The co-occurrence of peaks in the relative abundances of euplanktonic and tychoplanktonic species with peaks in the sand fraction detected in the lithostratigraphy indicate that a higher water table resulted from abrupt flooding. Regular rain events would have enabled the recurrence of more permanent water bodies and development of pelagic habitats. The presence of phytolith remains in samples containing diatoms further suggests that the environment surrounding the clay pan was at least temporally/partially covered by grass, in contrast to the present day barren landscape (Fig. 4). Overall, the sediment record clearly indicates a higher fluvial activity and sediment deposition during interglacial and interstadial stages (MIS 7, MIS 5, Figs 6 and 7) as compared to glacial/interglacial stages. A large interjacent hiatus, which we assign to MIS 6, indicates conditions of enhanced aridity in the catchment of the pan, by the absence of fluvial activity and deposition (Fig. 6).

## Regional climate records

We observe in our record an inverse correlation of wetter phases (Fig. 7) compared to high-resolution mid to late Pleistocene climate records from the Altiplano, Lake Titicaca, and adjacent lacustrine lakes and salars (Fig. 1A,B; e.g.^51–54^). Climate fluctuations in the Altiplano have been related to eccentricity-paced glacial-interglacial cycles, with glacials generally characterized by higher water input in lacustrine systems, whereas interglacials are generally indicated by drier conditions (Fig. 7; e.g.^51–54^). These changes coincide with variations in the intensity of the South American summer monsoon^53^. Furthermore, a causing factor for changing lacustrine conditions can be related to ‘El Nino Southern Oscillation’ (ENSO) changes, where warm events (El Niño-like conditions) supress moisture transport over much of the Altiplano^26^. Whereas the PAG record implies wetter phases in the Coastal Cordillera during MIS 7 and 5e, the Altiplano synchronously experienced pronounced arid conditions (Fig. 7).In contrast, during MIS 4 to MIS 1, aridity in the Coastal Cordillera was more enhanced while wetter conditions prevailed in the Altiplano, including large lake phases^51–54^ (Fig. 7).

A recent compilation of paleowetlands and paleolakes, covering the last <50 ka, from the Central Depression^25^, just a few 10 s of km away to the east (Fig. 1A), reveals a similar pattern and dependency on wetter episodes in the Altiplano, especially of the Central Andean Pluvial Events (CAPE, 17.5–14.2 ka, 13.8–9.7 ka^25^). Those paleowetlands were groundwater-fed by precipitation collected in the high Andes to the east^25^. The Coastal Cordillera is mostly hydrologically disconnected from the Central Depression and thus ground and surface water sourced from the Andes to the east does not reach Coastal Cordillera drainage systems. Despite the near proximity of our Coastal Cordilleran record and paleowetlands in the Central Depression^25^ (Fig. 1A), we cannot observe any significant fluvial activity in the Coastal Cordillera during the CAPE, which further underlines the particular position of our record within the Costal Cordillera and its valuable potential to record the regional climate of the hyperarid core. Wetter stages in the Coastal Cordillera must be rather controlled by moisture advection, which could reach the Coastal Cordillera and would provide enough precipitation within closed and smaller catchments therein. This setting can explain most likely the opposite trend found of arid/wet episodes in the Coastal Cordillera compared to those of the Altiplano and Central Depression (Figs 1A and 7). We conclude that the paleoclimate signal in the Coastal Cordillera, indicates past autochthonous precipitation signals.

Comparing our record to near-shore and distal marine drill-cores^28,55,56^, some dependencies become apparent (Figs 1B and 7). Stuut and Lamy^57^ reconstructed, based on grain size end-member data, a paleo-aridity index for northern Chile (27°S). They relate changes in continental aridity to changes in the latitudinal position of moisture-bearing Southern Westerlies, which could be driven by sea-ice extent around Antarctica and stimulated by tropical forcing in the equatorial Pacific Ocean. Strong rainfall events (positive index) are related to the occurrence of El Niño-like conditions, due to a weakening and northward displacement of the SE Pacific anticyclone^58^. Alternating wetter episodes during MIS5 reconstructed by Stuut and Lamy^57^ cannot be resolved in our record, where the entire MIS 5 is characterized by higher fluvial activity (Fig. 7). Due to the low temporal resolution of our record, short-term episodes of higher fluvial activity cannot be resolved with accuracy. We cannot observe any similarities between the aridity index and our record during the time period from MIS 4-MIS 1. Thus, we conclude that the aridity index from Stuut and Lamy^57^ does not represent the paleoclimate conditions for the hyperarid section of the Coastal Cordillera.

Marine records from offshore Peru and Ecuador (ODP1239, ODP849, ODP1237, TG7 Fig. 1B^59–62^), cover the last 225 ka (Fig. 7). Rincón‐Martínez, *et al*.^59^ used paleo sea surface temperatures (SST) and sedimentological proxies, to identify a predominance of El Niño-like conditions off the coast of Ecuador during interglacial times with higher continental moisture advection and runoff; whereas glacial times are characterized by predominantly La Niña-like conditions. Similar SST reconstruction were published by Calvo, *et al*.^61^ from the more southern Nazca ridge (17°S). Warmer SST can be connected to a warmer East Equatorial Pacific cold tongue and a southward shift of the equatorial front ITCZ system. A ‘rainy’ northern Atacama Desert was inferred using terrigenous biomarkers from marine sediments off Peru OPD 1229, 11°S^56^. Aeolian dust input into the near-shore Pacific Ocean off Peru ODP 1237^60^, and in the equatorial Pacific ODP 849^62^, indicates higher dust availability and deposition during glacial times (Fig. 7^60^). The fluctuations in the extent of arid and barren surface areas as sources of aeolian dust, accompanied with a meridional shift of the atmospheric circulation system in the Southeast Pacific, can be seen as the driving force for the variability in dust input in the East Pacific. Comparison of SSTs off Peru and Ecuador^59,61^ with our record, reveals a close synchronicity of rising SSTs and enhanced water availability in our study area. (Fig. 7). Higher SSTs are reconstructed for MIS 7, the onset of MIS 5e and most of the remaining MIS 5, accompanied by a sharp drop during MIS 4 and an increase towards the transition from MIS 3 to MIS 2. Only the most recent increase in SSTs during the Holocene is not preserved in our record (Fig. 7). Decreasing aeolian dust deposition in the East Pacific during MIS 7, onset and remaining of MIS 5^60^ can be connected to reconstructed wetter episodes in the Coastal Cordillera and higher SSTs in the East Pacific^59^. Recent climate models, simulating past precipitation events in the Atacama Desert, which controlled the floods in 2015, indicate that both, the northward moving cut-off low systems, and the existence of anomalous high SSTs off the coast of Chile are instrumental for high-intensity precipitation events^19^.

The paleoclimate reconstruction for the Coastal Cordillera indicates an opposite trend of moisture availability compared to continental Atacama/Altiplano records, which are mostly influenced by moisture advection from Atlantic air masses and precipitation in the high Andes (Fig. 7). This shows the separation of moisture sources affecting the Coastal Cordillera drainage systems. Coincidence with paleoclimate records from the marine Pacific, points to a connection of ‘wetter’ episodes in the Coastal Cordillera with increased SSTs off Chile/Peru during interglacial times (Fig. 7). Our reconstruction indicates that moist periods, characterized by enhanced fluvial activity within the catchment that carries coarse sand into an ephemeral lake, predominantly occurred during interglacial times, whereas dry periods, with subdued sedimentation of fine sediments took place during glacial times. The glacial-interglacial alternation during MIS 7 and MIS 5 mimics an eccentricity paced control on moisture in the Coastal Cordillera (Fig. 7).

## Conclusion

For the first time, we provide a paleoclimate record of the hyperarid core of the Atacama Desert from a clay pan in the Coastal Cordillera spanning the last 215 ka. Within the limits of the age model, the paleoclimate reconstruction points to wetter episodes with higher fluvial activity during MIS 7 and MIS 5 and reduced fluvial activity since MIS 4. Wetter periods in this section of the Coastal Cordillera are largely synchronous with enhanced East Pacific SST (conditions similar to modern ‘El Niño’), but are asynchronous with wet periods in the Altiplano, which can be explained by the different moisture sources (Pacific vs. Atlantic). During drier, glacial times low but still present sedimentation of mostly fine-grained material occurs, demonstrating that also during hyperarid conditions there is some surface activity in the study area, probably related to single rain events. Exceptions are large parts of the MIS 6 glacial period, when the absence of fluvial activity in the catchment caused a hiatus of more than 50 kyr in the record.

## Methods

To determine the sediment thickness in the terminal basin we conducted a GPR survey using a 100 MHz antenna with GSSI SIR-3000 system. For deeper penetration we utilized H/V ambient-noise seismometry. During two field campaigns in 2014 and 2015 two overlapping sediment cores (PAG 5, 6), with a maximum length of 6.2 m, were recovered using hand-held percussion drilling system (Eijkelkamp). Sediment cores were retrieved in opaque plastic liners. The unopened cores were analysed for their paleomagnetic properties, including inclination, declination and relative paleo-intensity, at LIAG (Grubenhagen). The elemental composition of the samples was determined using a ITRAX XRF core scanner (Cox Analytical Systems) at the University of Cologne and subsequently subsampled with a 2 cm resolution. Magnetic susceptibility was measured using a Kappa-Bridge KLY2 (Agico) sensor. GSD (<2 mm) were analysed, after a cation exchange pre-treatment to correct for gypsum, using a Beckman Coulter LS13320 laser particle sizer and evaluated using GRADISTAT^63^. Coarser grain sizes (>2 mm) were quantitatively affiliated. Diatom analysis was performed on 28 sediment samples. The age-depth model was obtained combining OSL (University of Cologne) and paleomagnetic data using AnalySeries V. 2.0^47^; and Bayesian statistics using ‘Bacon’^48^. Further information and details on the applied methods are provided in the supplementary dataset.

## Supplementary information

Supplementary
