# Supplementary material for: “Climatic fluctuations in the hyperarid core of the Atacama Desert during the past 215 ka”
Source: Sci Rep. 2019 Mar 27;9:5270. doi: 10.1038/s41598-019-41743-8 (PMC6437205; doi:10.1038/s41598-019-41743-8)
Supplement: Supplementary file 1 — Supplementary [file 41598_2019_41743_MOESM1_ESM.pdf]

Supplementary Material

"Climatic fluctuations in the hyperarid core of the Atacama Desert during the past 215 ka"

Benedikt Ritter [1], Volker Wennrich [1], Alicia Medialdea [2], Dominik Brill [2], Georgina King [3], Sascha Schneiderwind [4], Karin Niemann [4], Emma Fernández-Galego [1], Julia Diederich [1], C. Rolf [5], Roberto Bao [6], Martin Melles [1], Tibor J. Dunai [1]

1 Institute of Geology & Mineralogy – University of Cologne, Cologne, Germany

2 Institute of Geography – University of Cologne, Cologne, Germany

3 Institute of Earth Surface Dynamics – University of Lausanne, Lausanne, Switzerland

4 Institute of Neotectonics and Natural Hazards - RWTH Aachen University, Aachen, Germany

5 Leibniz Institute for Applied Geophysics (LIAG), Hannover, Germany

6 Centro de Investigacións Científicas Avanzadas (CICA), Facultade de Ciencias, Universidade da Coruña, Spain

*Correspondence to benedikt.ritter@uni-koeln.de

1. Methods

1.1. Geophysical Site Survey

At the terminal pan, 35 ground-penetrating radar (GPR) profiles extending to approx. 23 km were obtained using a GSSI 100 MHz antenna (+survey wheel) and a GSSI SIR-3000 system. For spatial referencing the profiles were GPS-tracked. Data pre-processing using the REFLEXW ® software Version 7.2, [1], includes tools to enhance visibility of subsurface structures. In general, the processing includes start time correction, background removal, manual gain adjustments, and 1D-filtering followed by topographic correction. Afterwards, transition layers were picked in each profile and stored as xyz-point data, where x and y represent global UTM coordinates and z is the layer depth delineated by the two-way-travel time of the electromagnetic waves and their velocity in the ground. The point data depict measurements of a surface that can be estimated by interpolation functions provided in the Matlab Curve Fitting ToolboxTM including outlier removal, splines, and biharmonic fitting. The resulting surface function was applied to an artificial point cloud dataset with point spacing of 5 m and for a better visualisation transformed into a raster format of 5 m resolution in ArcGIS.

For further estimation of total sediment thickness and bedrock contact of the basin, the horizontal-to-vertical (H/V) ambient-noise seismic method was applied, which was originally proposed by Nogoshi and Igarashi [2] and widely applied by Nakamura [3]. A seismic profile (NNW-SSW) with up to 16 noise measurements across the clay pan was conducted. Recording the resonance frequencies of basin deposits with a Lennartz LE3D5s seismometer and a LEAS City Shark II data logger was carried out with a 200 Hz

sampling rate and 30 min sampling duration. In order to avoid disturbances by external influences (wind), the seismometer was installed in a small hole.

Processing steps and peak evaluation using GEOPSY processing software were carried out according to Acerra, et al. [4]. H/V ratios were calculated from time windows of min. 20 s to max. 40 s length extracted from raw data. Calculation of soft-layer thickness (h) for each measurement was made on the assumption that resonance occurs at frequencies (f_r), where uneven multiples of λ/4 fit the layer's thickness (h). For constant S-wave velocity in sediment layers' resonance occurs at:

$$f_r = \frac{v_{S,clay}}{4 * h_r}$$

h_r depth of transition between hard and soft rock [m]

$v_{S,clay}$ approx. shear wave velocity in clay (≈ 300 m/s) [m/s]

f_r fundamental frequency (derived by H/V ratios) [Hz]

Calibrating with GPR measurements to approximate average shear wave velocity $v_{S,clay}$ failed due to hard cover of the first few cm of sediments and low impedance contrasts between sediment layers. An approximation of shear wave velocity with respect to a reference value [5,6] was used. Calculation of h was done according to (z = altitude):

$$h = z - h_r.$$

1.2. Drilling

In 2014 and 2015 the uppermost 6.2 m (Composite of PAG 5 and 6) of the sediment record in the central part of the clay pan have been recovered by hand-held percussion drilling system (Eijkelkamp). Opaque liners were used to avoid any UV radiation destroying the luminescence signal. Whole-core scanning was conducted at the University of Cologne (Multi-Sensor-Core Logger for magnetic susceptibility for further paleomagnetic analysis, Institute of Geology & Mineralogy) and at the Leibniz Institute for Applied Geophysics (Paleomagnetic). Subsampling was carried out with a sample resolution of 2 cm. Gaps in the sediment record are due to recovery loss during drilling.

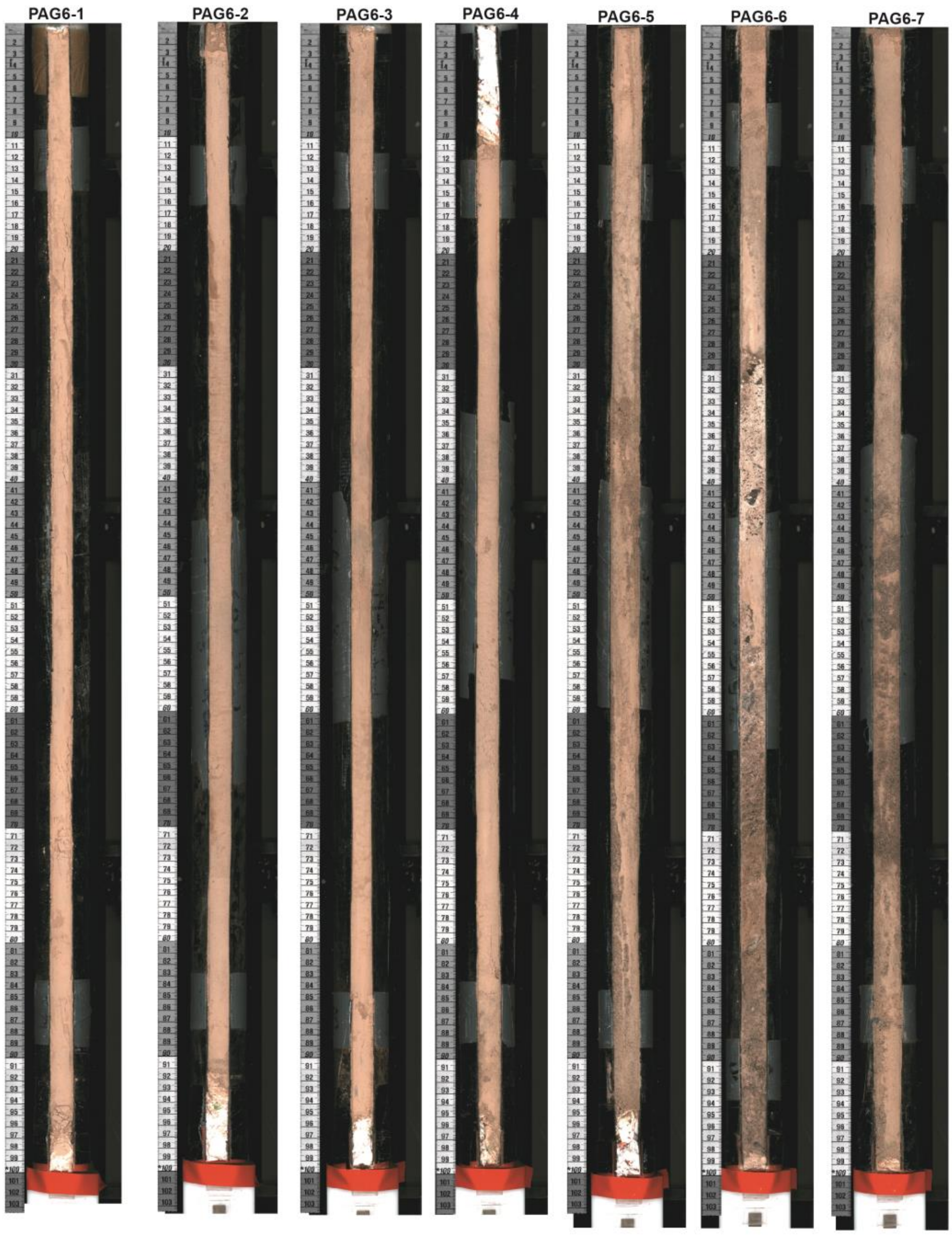

Fig. S1: Sediment core images of PAG6.

1.3. Inorganic Geochemistry and Physical Properties

ITRAX (Cox AnalyticalSystems, Sweden) XRF scanning was conducted to gain information about the inorganic geochemistry the sediment record. The X-ray generator used in this study provides 1.9 kW (Cr-tube). Each sample was measured with 30 kV and 55 mA. Data processing was performed with the software QSpec 6.5 (Cox Analytical Systems, Sweden). Relative element amounts are represented by counts per 20 second [7].

Magnetic Susceptibility (MS) was measured on 2 cm paleomagnetic sample cubes (PAG5, 6) using a Kappa-Bridge KLY2 (Agico). Grain-size analysis was performed at a 2 cm resolution, on aliquots of 1.0 g. Samples were first sieved (dry) by 2 mm and 6.3 mm meshes, with subsequent weighing of the resulting gravel (>2 mm) and finer (<2 mm) fraction. In general, terrigenous sediments of depositional systems in arid to hyperarid climates can be considered as a mixture of aeolian input, fluvial deposits, lacustrine products and evaporitic precipitates. In order to detect the grain-size distributions of past

allochthonous sediment input, autochthonous evaporitic and lake products were erased by chemical treatment. For the removal of gypsum, the sub-sample <2 mm was treated with sodium bicarbonate solution (210 g/l, 12 h at 60°C). This treatment converts the gypsum to calcium carbonate, which is subsequently dissolved by hydrochloric acid (HCl, 10 %). Prior to grain-size analysis, the samples were dispersed on a shaker for 12 h and underwent one minute of ultrasonic treatment. Sample aliquots were measured three times with a Beckman Coulter LS13320 laser particle sizer equipped with an autosampler, and the individual results were averaged. Grain-size data, including standard parameters, such as sorting (σ), skewness (SK), or kurtosis (K) were evaluated applying logarithmic graphic measures from Folk and Ward [8] and using GRADISTAT [9]. Grain-size fractions are given as percentages of the bulk sample (including gravel fraction).

1.4. Diatom Analysis

For diatom analyses, which were carried out on 28 samples sediment samples were treated following standard procedures of acid digestion with hydrochloric acid and hydrogen peroxide, and then washed repeatedly with distilled water [10]. A small fraction of each sample was then evaporated onto glass coverslips and mounted onto permanent slides using the high refractive index medium Naphrax. The diatoms on the slides were counted along parallel transects at X1000 magnification using a Nikon E600 microscope equipped with differential interference contrast optics. Diatom concentration was extremely low, but at least 100 valves were counted per sample (average of 228 valves per sample). Taxonomic identification was based upon the available diatom floras from the region and elsewhere[11-17].

1.5. Age-Depth Model

Due to the absence of organic remains in the core, excluding radiocarbon dating, we used a combination of Optically Stimulated Luminescence (OSL) and paleomagnetic dating to obtain a reliable age-depth model. The composite core chronology was derived using AnalySeries V. 2.0 [18] for a preliminary age-depth model. Final age-depth modelling was performed using the Bayesian age-depth modelling software 'rBacon' of Blaauw and Christen [19]. Periods of non-deposition or erosion are included into the model as hiatuses.

1.5.1. Optically stimulated luminescence (OSL) dating

OSL dating was carried out at the Cologne Luminescence Laboratory (CLL) on twelve fine-grained sediment samples from the uppermost 6 m of cores PAG 1 and 6. Samples for burial dose determination were taken from closed cores opened under subdued red light conditions. They were pre-treated following standard procedures to extract the polymineral 4-11 µm grain-size fraction [20]. Aliquots of this grain-size fraction were mounted from suspension onto 9.8 mm diameter stainless steel discs. Measurements were done using conventional Risø TL/OSL readers with $^{90}Sr/^{90}Y$ beta sources delivering between 0.10 and 0.13 Gy/s at the sample position. Signals were stimulated using infrared diodes (870±40 nm), and detected through a 410 nm interference filter. Equivalent dose measurements were performed using the post-infrared infrared protocol measured at 225 °C, pIRIR225.

Aliquots bleached for 24 hours in a solar simulator were used to determine residual signals and to test the reproducibility of the protocol by means of a dose-recovery test. Experiments were conducted following Auclair, et al. [21] to measure rates of anomalous fading. For each sample 6-30 aliquots were measured. Derived dose values, D_e, were accepted if the recycling ratio was between 0.9 and 1.1 and recuperation was <5 % of the

measured D_e. Over-dispersion of the dose distributions, OD, were <8% in all cases. Measurement of fine-grained aliquots containing thousands of grains provides an average dose to which well and incompletely bleached grains contribute, i.e. grains with insufficient exposure to light during transport prior to deposition. This effect is widespread in fluvial settings, especially in arid regions, where high sediment load and rapid transport during short intense rain events may lead to incomplete bleaching [22]. It is therefore expected that if the sediment is affected by incomplete bleaching, the average D_e values of fine-grained aliquots, which average the luminescence signal of up to 1×10^6 grains, might overestimate the true burial dose and the derived age. Incomplete bleaching of polymineral fine-grained samples can be evaluated through comparison between the pIR225 signal and the more easily bleached IR50 signal (e.g. [23-25]). This has been checked for all samples deriving in an IR50 to pIR225 average ratio of ~0.7, suggesting that the sediments are not affected by incomplete bleaching.

The central age model [26] was used for burial-dose calculation. Dose rate and age calculation was done using DRAC v1.2 [27]. Dose rates are based on radionuclide concentrations (U, Th, K) in the surrounding sediment derived from high-resolution gamma spectrometry. In situ water contents (the modern aridity is assumed to be representative for most of the burial period), geographical position and sediment overburden were taken into account in the calculation. An internal K content of 12.5±0.5 %, adopted from Huntley and Baril [28], and an a-value of 0.11±0.02 [29] to account for the contribution of alpha radiation, have been assumed.

1.5.2. Paleomagnetic - paleo-inclination & paleo-intensity reconstruction

The acquisition of magnetostratigraphic data, i.e., inclination, declination, and paleo-intensity was performed on whole-core samples running through a pass-through cryogenic magnetometer from 2-G Enterprises [30] with an embedded AF demagnetizer (max. 30 mT) at the Leibniz Institute for Applied Geophysics (LIAG) in Grubenhagen, Germany. The natural remanent magnetization (NRM) was determined after stepwise demagnetization (0, 10, 20, 30 mT) following the procedure after Rolf [30] and Rolf, et al. [31]. The relative paleo-intensity of the sediments was calculated using the ratio between the Natural Remanent Magnetization (NRM) at 30 mT to the magnetic susceptibility (MS) with the MS acquired in 2-cm resolution with a Bartington MS2E sensor on a multi-sensor core logger (MSCL-S; Geotek Ltd.).

2. Results

2.1. Lithological Characterisation

Grain Size Distributions

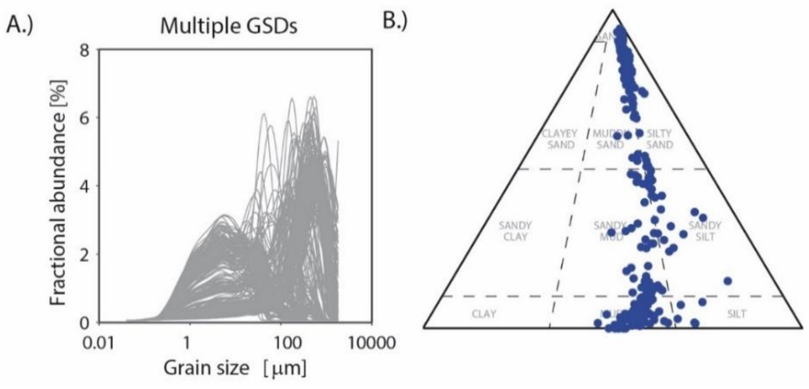

Figure S2: A.) Multi-Specimen grain size spectra plot B.) Sample distribution in a clay-silt-sand diagram with sediment classification according to after Folk Folk [32] and Shepard Shepard [33]. Plots created by AnalySize [34].

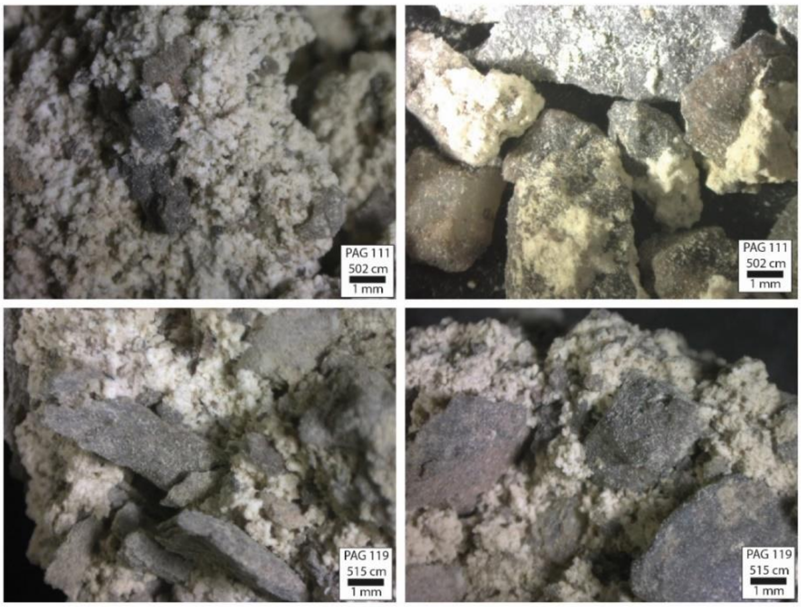

Figure S3: Photographs of some sediment samples from the gypsum crust sequence (~500-525 cm). Clasts are surrounded and cemented by calcium-sulphate, finer grain-sizes are mostly vanished. Gypsum partly appears as nodules.

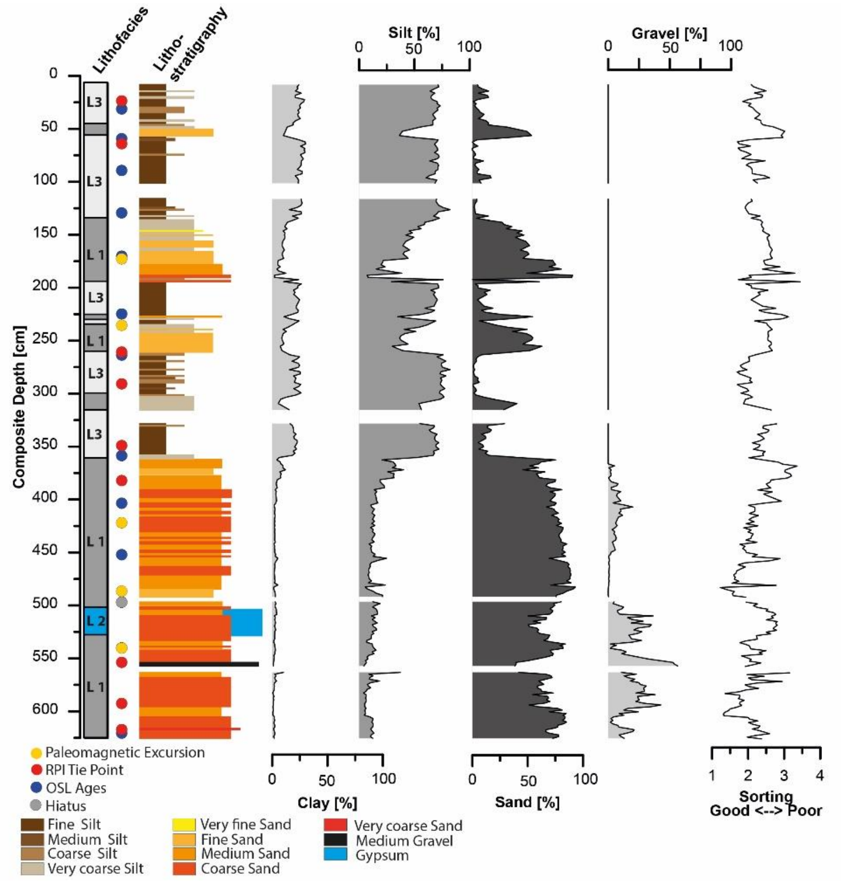

Fig. S4: Lithofacies and lithology based on grain-size distributions (0->6.3 mm). Grain-size plots based on Beckman Coulter data (0-2 mm) and weighted gravel fraction (>2 mm). Sorting of the <2 mm fraction is based on laser particle size data. Coloured dots mark age tie points, see age-depth model (Fig. S6).

2.2. Biostratigraphical Characterisation

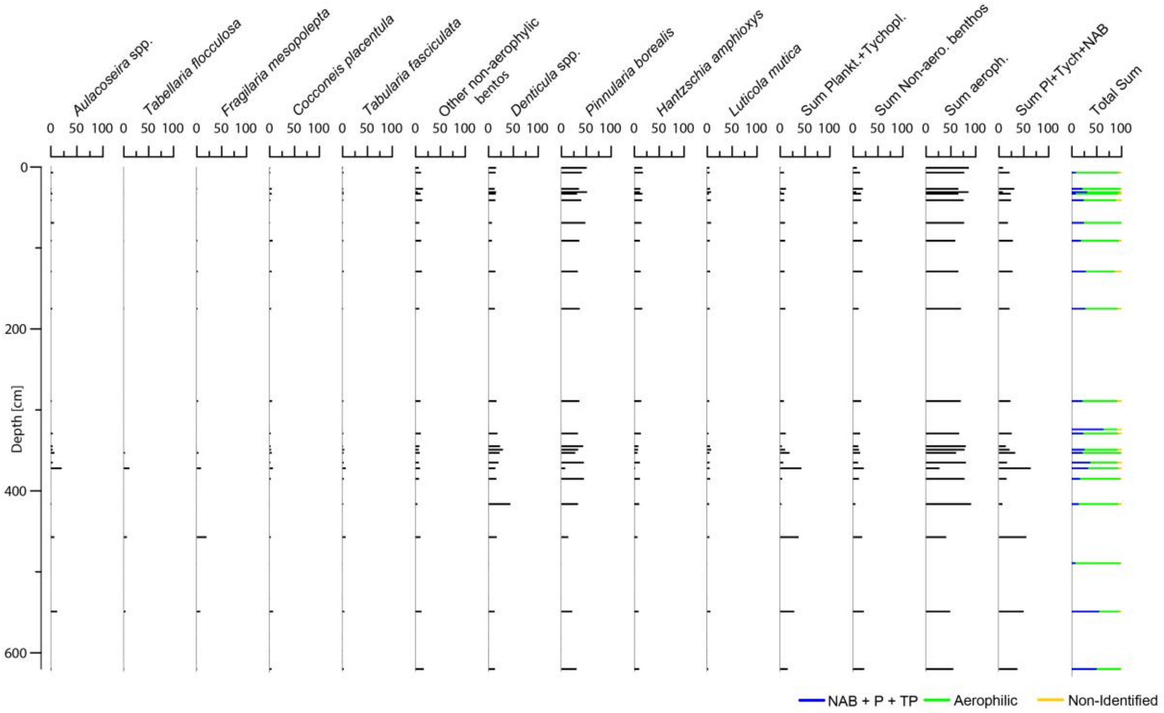

Figure S5: Simplified diatom percentage diagram for PAG5, 6.

2.3. Core Chronology:

Table S1: Radionuclide data and total environmental dose rate. Water content 0 ±3 %, depth uncertainty ±0.05 m.

Sample	Depth (m)	U (ppm)	Th (ppm)	K (%)	Total environmental dose rate (Gy/ka)
**PAG I-2**	0.32	*2.45* ± *0.13*	*9.73* ± *0.60*	*2.08* ± *0.02*	5.26 ± 0.23
**PAG I-3**	0.6	*2.49* ± *0.14*	*9.62* ± *0.58*	*2.02* ± *0.02*	5.17 ± 0.23
**PAG I-4**	0.9	*2.46* ± *0.14*	*9.65* ± *0.58*	*2.14* ± *0.03*	5.27 ± 0.23
**PAG I-5**	1.3	*2.50* ± *0.20*	*9.50* ± *0.60*	*2.10* ± *0.10*	5.22 ± 0.24
**PAG 6-2**	1.7	*2.14* ± *0.12*	*8.14* ± *0.49*	*1.68* ± *0.02*	4.38 ± 0.20
**PAG 6-3a**	2.25	*2.23* ± *0.12*	*9.04* ± *0.54*	*1.97* ± *0.02*	4.85 ± 0.21
**PAG 6-3b**	2.64	*2.23* ± *0.12*	*9.04* ± *0.54*	*1.97* ± *0.02*	4.85 ± 0.21
**PAG 6-4b**	3.59	*2.40* ± *0.40*	*7.40* ± *0.50*	*1.78* ± *0.14*	4.47 ± 0.24
**PAG 6-5a**	4.03	*2.20* ± *0.30*	*6.50* ± *0.40*	*1.46* ± *0.09*	3.89 ± 0.20
**PAG 6-5b**	4.52	*1.53* ± *0.26*	*6.60* ± *0.40*	*1.43* ± *0.12*	3.50 ± 0.17
**PAG 6-6b**	5.40	*1.90* ± *0.09*	*7.22* ± *0.37*	*1.54* ± *0.03*	3.90 ± 0.17
**PAG 6-7b**	6.20	*1.66* ± *0.08*	*6.30* ± *0.32*	*1.48* ± *0.03*	3.55 ± 0.15

Table S2: Age estimates for geomagnetic excursion covering the last 215 ka. Given depths and ages are used to calculate an age model. Ages are given related to the occurrence of each geomagnetic excursion in the PISO1500 stack [35].

**References for Paleomagnetic Excursions**				**PISO1500**	**This Study**
	**Age [ka]**	**Uncertainty [ka]**	**Reference**	**Age [ka]**	**Depth[cm]**
**Mono Lake / 3α**	33	1	Lund, et al. [36]	**34**	**174**
	33	1	Channell [37]		
**Laschamp / 3β**	41	1	Lund, et al. [36]	**41**	**236**
	41	1	Channell [37]		
	40.9	1	Channell, et al. [38]		
	41.3	0.6	Laj, et al. [39]		
**Post Blake Event 5α**	~100		Lund, et al. [36]	**99**	**422**
	**98**	**2**	Channell, et al. [35]		
**Blake / 5β**	123	3	Lund, et al. [36]	**120**	**487**
	**120.5**	**1.5**	Channell, et al. [35]		
	120	12	Singer, et al. [40]		
**Iceland Basin / 7α**	~190		Lund, et al. [36]	**194**	**554**
	184	4	Channell [37]		
	188	3.5	Channell, et al. [38]		
	**190.2**	**1.77**	Channell [41]		
**Pringle Falls**	~211		Laj & Channell[42]		
	**211**	**1.5**	Roberts et al. [43]		

Age-Depth Models

1. Age-Depth Model Input Parameter:

This chronology includes geomagnetic excursions indicated in Fig. S6a (including Post-Blake Event), the uppermost seven OSL ages and additionally eight RPI tie points (Fig. S6b). OSL ages (PAG 6-4b, PAG 6-5a, and PAG 6-5b) were not used/included in the age-depth modelling using a Bayesian statistic approach ('rBacon' [19]). The OSL signals of these samples may not have been fully reset during transport of the coarser sediments found below 360 cm. Two high sedimentation rate episodes (according to OSL and geomagnetic data) between 131-191 cm and 236-264 cm (coarse layers) were treated as event layers. The pedogenic gypsum layer (500-525 cm) was treated as a hiatus at the top of Lithofacies 2. The applied models were modified using following parameters acc.mean=500, acc.shape=1.5, mem.mean=0.4 and mem.strength=6. Age-depth models were merged (transition hiatus at 500 cm) as the combination of event layers and a hiatus resulted in anomalous non-realistic age models and/or one of the features were not included into the Bayesian statistics. Accumulation time plot was calculated using a model without event-layers.

2. Age-Depth Model Input Parameter:

Following the suggestion of one reviewer we developed an alternative age-depth model. This alternative chronology assumes that the Post-Blake Event at 420 cm is assigned to the Blake Event (~120 ka) instead. This results in a shift of assigned geomagnetic excursion and to the inclusion of the Pringle Falls event at ~211 ka. All OSL ages (except of the stratigraphically lowest two OSL ages, PAG 6-6b, PAG 6-7b; which are saturated)

were included into the age-depth model. Additionally, we identified four potential RPI tie points to this chronology. Identical to the first age model, the two episodes of higher sedimentation were included as event layers into the model. Applied models were modified using following parameters acc.mean=500, acc.shape=1.5, mem.mean=0.3 and mem.strength=7. Age-depth models using assumed hiata at either 454 or 484 cm, had to be calculated and merged (with the model without hiatus). Age-depth modelling of all features failed in one run due to anomalous age-depth model results. The age-depth model assuming no hiatus indicates an episode of high accumulation time, which coincide with high probability to the occurrence of a hiatus between 454-484 cm. Due to this finding, two models with a minimum hiatus at 454 cm and a maximum hiatus at 484 cm were included in the modelling processes to account for the effects of potential hiata. Accumulation time plot was calculated using a model without event-layers.

The alternative assignment of one geomagnetic excursions at 422 cm to the Blake Event (120.5 +/-1.7 ka) and a subsequently shift of geomagnetic excursions would fit the measured OSL ages (Fig. S6D). The corresponding switch of the geomagnetic excursions at 487 cm (Iceland Basin 190 ± 5 ka) and at 554 cm (Pringle Falls 211 ± 1.5 ka) would slightly change the age-depth model to older modelled ages. This scenario indicates, compared to the first age-depth model, an episode of reduced accumulation into the basin between 454-484 cm (~150-190 ka). However, this modelled hiatus or episode of low accumulation is not reflected in the observed sedimentology. We modelled a younger hiatus at 454 cm (hiatus ~150-174 ka) and an older hiatus at 484 cm (hiatus ~163-189 ka) to indicate the potential chronological effects of non-sedimentation (Fig. S6D). The potential occurrence of a hiatus partly coincides with the assumed hiatus of our chronology (total potential duration ~150-180 ka). However, this age chronology indicates certain disagreements with sedimentological data. The occurrence of the gypsum crust, as indicator for surface stagnancy would be shifted into a time of higher more stable sedimentation, which clearly contradicts the paleo-environmental reconstruction and evolution of a gypsum crust. Additionally, this period of low to non-sedimentation would coincide with the highest planktonic diatoms occurrence we could identify in the record, which would contradict the paleo-environmental reconstruction related to the presence of planktonic diatoms and lacustrine conditions.

Both age-depth models indicate an occurrence of a hiatus between ~135-190 ka, covering large parts of the MIS 6 glacial. The preferred age-depth model (including Post-Blake) indicates a maximum age at the bottom of ~215 ka. Sedimentation of coarser sediments during ~215-180 ka and ~135-70 ka with the occurrence of a gypsum-crust and concomitantly hiatus from ~180-135 ka (Fig. S6C). Accumulation of finer grain sizes take place between ~70-6 ka. The age-depth model indicates a maximum age of ~242 ka at the bottom, and phases of coarser sedimentation between ~242-205 ka and ~194-90 ka, with the occurrence of the gypsum crust between ~205-194 ka (Fig. S6D). Calculated sedimentation rates between ~189-150 ka are very low and point to the occurrence of an untraceable hiatus. Sedimentation of finer grain sizes into the basin would have occurred between ~90-6 ka. Both age-depth models have either discrepancy with the OSL ages or the sedimentological record. We place our confidence in the sedimentary record. The age differences between both models is approximately/maximum 10-30 ka between ~300-500 cm and 10-20 ka between 500-624 cm, respectively. We note that irrespective of which age-depth model is applied the main conclusion of our study, asynchonicity of wet/dry phases in the Altiplano and Coastal Cordillera remains.

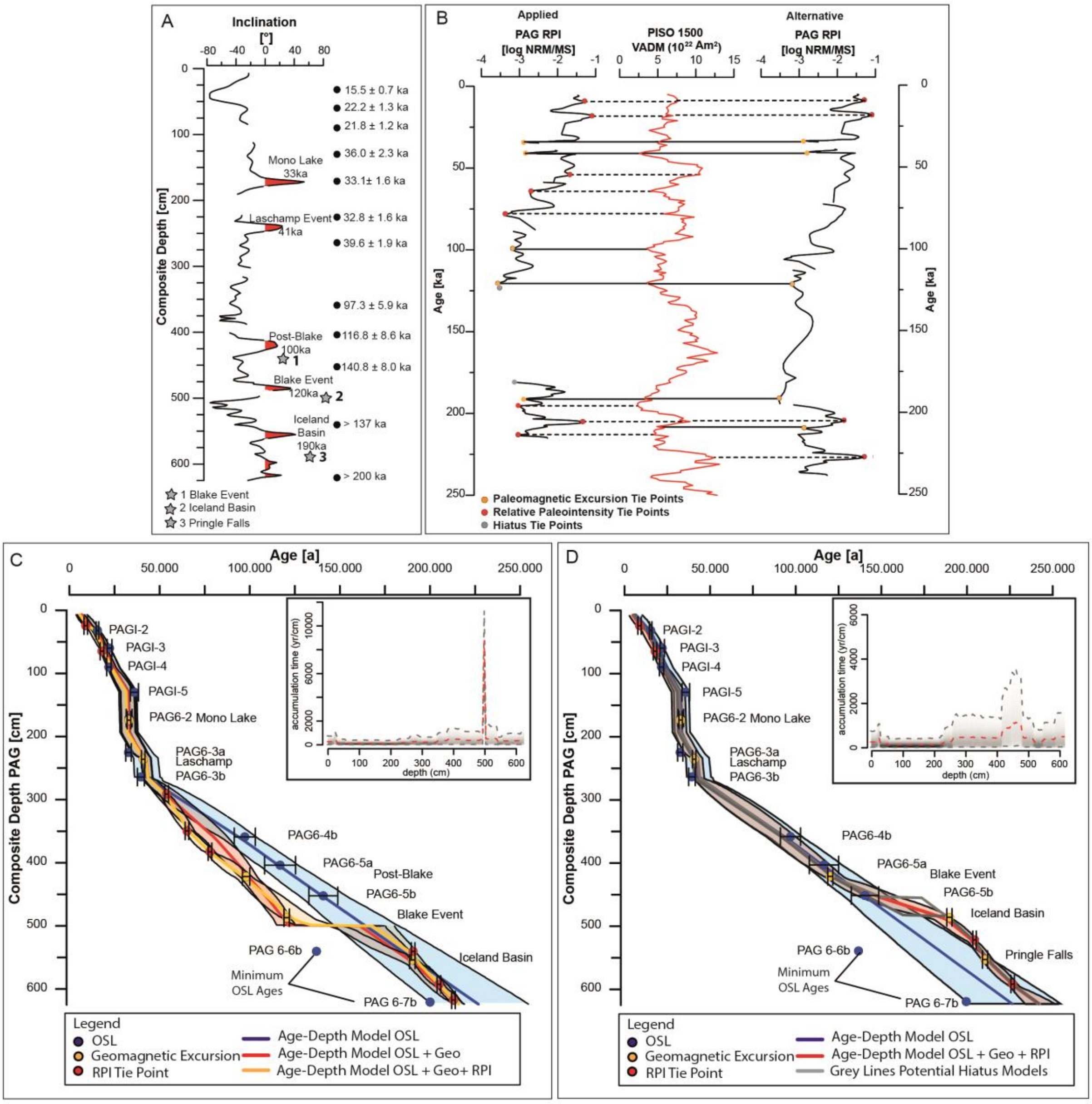

Figure S6 (A) Paleomagnetic inclination record of core PAG6. Geomagnetic inclination excursion recorded in the sediment record are marked in red with proposed geomagnetic events. Grey stars indicate an alternative assignment of potential geomagnetic excursion. Black dots display measured OSL ages. (B) Relative paleointensity (RPI) record (applied chronology and alternative chronology) vs. used RPI stacks PISO1500 40. Orange dots and solid lines indicate geomagnetic excursion tie points, red dots and dashed lines mark RPI tie points. Grey dots mark tie points for resembling phases of sedimentation stagnancy (hiatus). (C) and (D) derived Bayesian age-depth models using 'Bacon' from Blaauw and Christen 48. Orange dots indicate geomagnetic excursion tie points, red dots RPI tie points and blue OSL ages. Accumulation time is given in yr/cm (calculated using 'Bacon'48 without event-layers). Note that abrupt changes in the sedimentation rate is tightly linked to used tie points and do not reflect the likely smooth natural transitions. The lowest OSL ages (PAG 6-6b, PAG 6-7b) represent minimum ages. The alternative age-depth model (D) indicates an episode of relatively low sedimentation/high accumulation time around 454-484 cm, however a direct assignment of a hiatus, based on sedimentology is not possible. To accommodate the potential episode of non-sedimentation, two additional age-depth

models were modelled for a minimum hiatus at 454 cm and a maximum hiatus at 484 cm (grey lines between 454-484 cm).

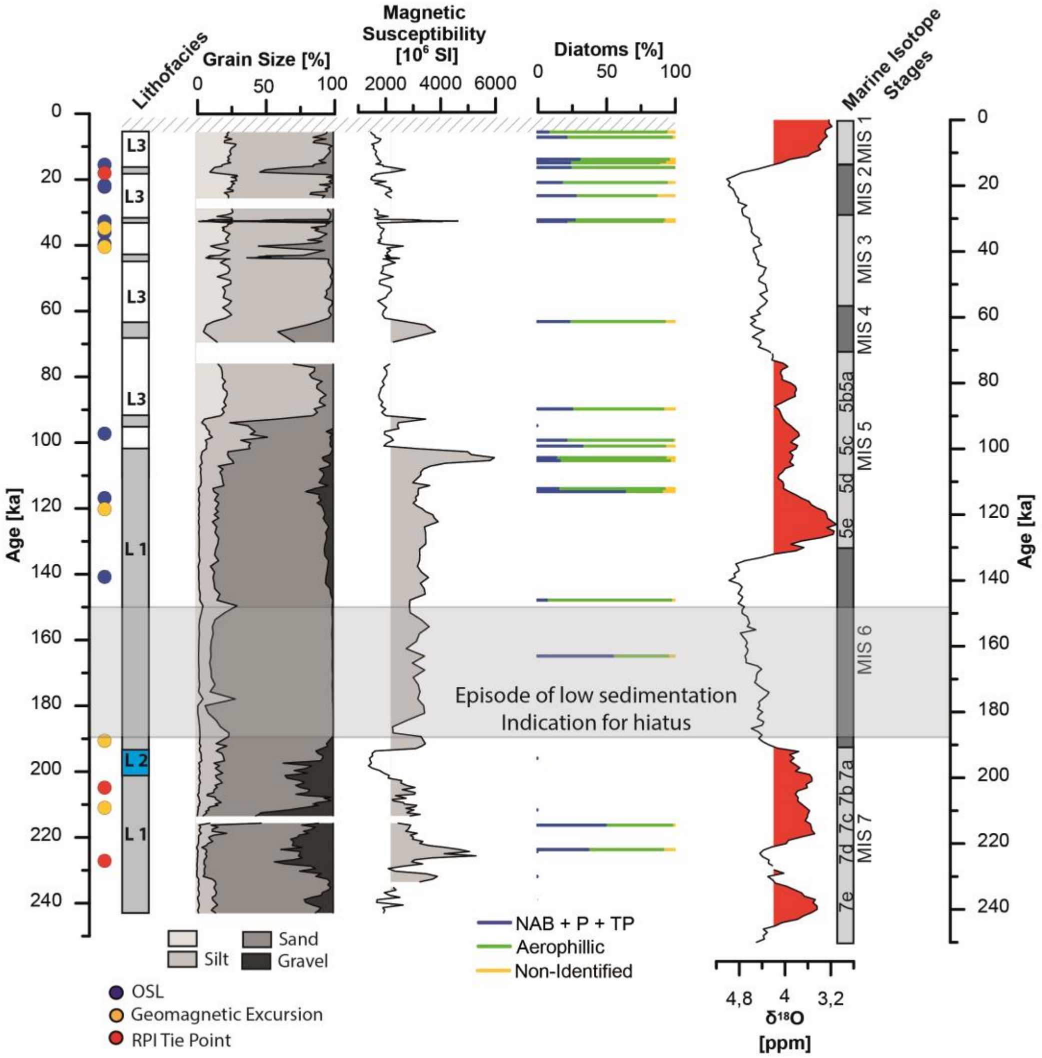

Fig. S7: Figure with identical format to figure 6 in manuscript, however, applying the alternative chronology. Note that interglacials are (MIS 7 and 5) are characterized by coarse (gravel) sediment deposition. Based on the alternative age-depth model a potential hiatus has to be taken into account between 150-180 ka. The latter episode is characterized by very low sedimentation rates and high accumulation times, so that the record is stretched. Note that the assumed hiatus represented by the pedogenic gypsum is placed in a period of higher sedimentation, when using the alternative chronology.

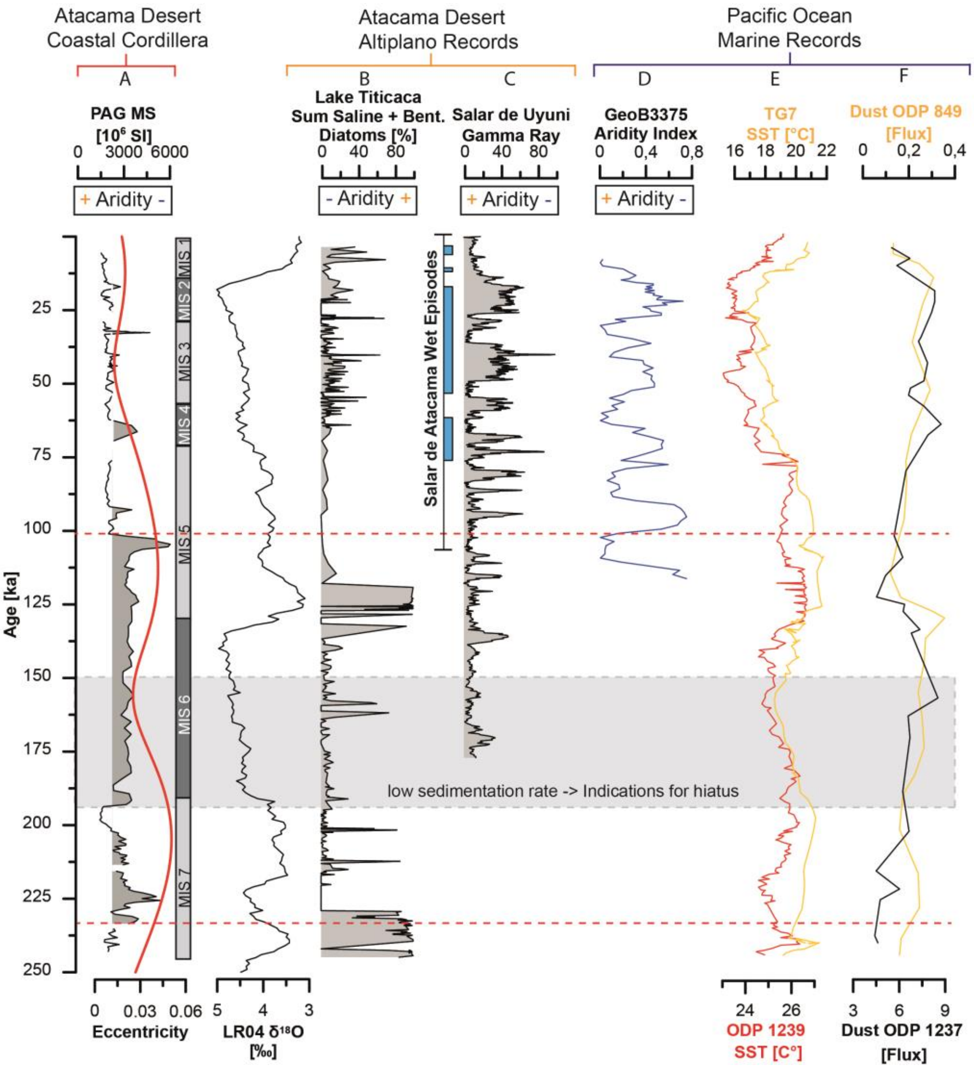

Fig. S8: Discussion figure 7 using the proposed alternative age-depth model. Note the grey marked area for an episode of low sedimentation rate / high accumulation time, indicative for the presence of hiatus between 454-484 cm.

References

1 Sandmeier, K. ReflexW v7. 5 Manual. *from avaliable: http://www. sandmeiergeo. de/reflexw. html* (2015).

2 Nogoshi, M. & Igarashi, T. On the amplitude characteristics of microtremor (part 2). *Jour. Seism. Soc. Japan* **24**, 26-40 (1971).

3 Nakamura, Y. A method for dynamic characteristics estimation of subsurface using microtremor on the ground surface. *Railway Technical Research Institute, Quarterly Reports* **30** (1989).

4 Acerra, C. *et al.* Guidelines for the implementation of the H/V spectral ratio technique on ambient vibrations measurements, processing and interpretation. (2004).

5 Bravo, R. Estudio geofisico de los suelos de fundación para un zonificacion sísmica del area urbana de Santiago Norte. *Memoria de título de Ingeniero Civil, Fac. Cs. Fis. y Mat., Universidad de Chile* (1992).

6 Guéguen, P. Microzonage de santiago du Chile (technique de Nakamura). *Master Thesis. Joseph Fourier University, Grenoble, France* (1994).

7 Croudace, I. W., Rindby, A. & Rothwell, R. G. ITRAX: description and evaluation of a new multi-function X-ray core scanner. *Geological Society, London, Special Publications* **267**, 51-63 (2006).

8 Folk, R. L. & Ward, W. C. Brazos River bar: a study in the significance of grain size parameters. *Journal of Sedimentary Research* **27** (1957).

9 Blott, S. J. & Pye, K. GRADISTAT: a grain size distribution and statistics package for the analysis of unconsolidated sediments. *Earth surface processes and Landforms* **26**, 1237-1248 (2001).

10 Battarbee, R. W. *et al. Diatoms*. (Springer, 2002).

11 Frenguelli, J. *Diatomee fossili delle conche saline del deserto cileno-boliviano*. (1928).

12 Frenguelli, J. *Diatomeas de la caliza de la cuenca de Calama en el desierto de Atacama Chile: Univ. nac. de la Plata. Inst. del Museo*. (Coni, 1936).

13 Rumrich, U. & Rumrich, M. *Diatomeen der Anden: von Venezuela bis Patagonien/Feuerland: und zwei weitere Beiträge*. Vol. 9 (ARG Gantner Verlag KG, 2000).

14 Servant Vildary, S. Les Diatomées des Sédiments superficiels d'un lac salé, Chloruré, sulfaté sodique de L'Altiplano Bolivien, le lac Poopó. *Cahiers ORSTOM. Série Géologie* **10**, 79-90 (1978).

15 Servant Vildary, S. & e Sousa, S. M. Palaeohydrology of the Quaternary saline Lake Ballivian (southern Bolivian Altiplano) based on diatom studies. *International Journal of Salt Lake Research* **2**, 69-85 (1993).

16 Krammer, K. Bacillariophyceae 1. *Susswasserflora von mitteleuropa* (1986).

17 Krammer, K. & Lange-Bertalot, H. 1991. Süsswasserflora von Mitteleuropa. Bacillariophyceae 1–4. *Stuttgart: Gustav Fisher* (1986).

18 Paillard, D., Labeyrie, L. & Yiou, P. AnalySeries 1.0: a Macintosh software for the analysis of geophysical time-series. *Eos* **77**, 379 (1996).

19 Blaauw, M. & Christen, J. A. Flexible paleoclimate age-depth models using an autoregressive gamma process. *Bayesian analysis* **6**, 457-474 (2011).

20 Zander, A. & Hilgers, A. Potential and limits of OSL, TT-OSL, IRSL and pIRIR(290) dating methods applied on a Middle Pleistocene sediment record of Lake El'gygytgyn, Russia. *Climate of the Past* **9**, 719-733, doi:10.5194/cp-9-719-2013 (2013).

21 Auclair, M., Lamothe, M. & Huot, S. Measurement of anomalous fading for feldspar IRSL using SAR. *Radiation measurements* **37**, 487-492 (2003).

22 Porat, N. *et al.* Abandonment ages of alluvial landforms in the hyperarid Negev determined by luminescence dating. *Journal of Arid Environments* **74**, 861-869 (2010).

23 Colarossi, D., Duller, G., Roberts, H., Tooth, S. & Lyons, R. Comparison of paired quartz OSL and feldspar post-IR IRSL dose distributions in poorly bleached fluvial sediments from South Africa. *Quaternary Geochronology* **30**, 233-238 (2015).

24 Kars, R. H., Reimann, T., Ankjærgaard, C. & Wallinga, J. Bleaching of the post-IR IRSL signal: new insights for feldspar luminescence dating. *Boreas* **43**, 780-791 (2014).

25 Buylaert, J.-P. *et al.* Luminescence dating of the PASADO core 5022-1D from Laguna Potrok Aike (Argentina) using IRSL signals from feldspar. *Quaternary Science Reviews* **71**, 70-80 (2013).

26 Galbraith, R. F., Roberts, R. G., Laslett, G. M., Yoshida, H. & Olley, J. M. Optical dating of single and multiple grains of quartz from Jinmium rock shelter, northern Australia: Part I, experimental design and statistical models. *Archaeometry* **41**, 339-364 (1999).

27 Durcan, J. A., King, G. E. & Duller, G. A. DRAC: Dose Rate and Age Calculator for trapped charge dating. *Quaternary Geochronology* **28**, 54-61 (2015).

28 Huntley, D. J. & Baril, M. The K content of the K-feldspars being measured in optical dating or in thermoluminescence dating. *Ancient TL* **15**, 11-13 (1997).

29 Kreutzer, S., Schmidt, C., DeWitt, R. & Fuchs, M. The a-value of polymineral fine grain samples measured with the post-IR IRSL protocol. *Radiation Measurements* **69**, 18-29 (2014).

30 Rolf, C. Das Kryogenmagnetometer im Magnetiklabor Grubenhagen. *Geologisches Jahrbuch* **E52**, 161-188 (2000).

31 Rolf, C., Hambach, U., Novothny, Á., Horváth, E. & Schnepp, E. Dating of a Last Glacial loess sequence by relative geomagnetic palaeointensity: a case study from the Middle Danube Basin (Süttő, Hungary). *Quaternary International* **319**, 99-108 (2014).

32 Folk, R. L. The distinction between grain size and mineral composition in sedimentary-rock nomenclature. *The Journal of Geology* **62**, 344-359 (1954).

33 Shepard, F. P. Nomenclature based on sand-silt-clay ratios. *Journal of Sedimentary Research* **24** (1954).

34 Paterson, G. A. & Heslop, D. New methods for unmixing sediment grain size data. *Geochemistry, Geophysics, Geosystems* **16**, 4494-4506 (2015).

35 Channell, J., Xuan, C. & Hodell, D. Stacking paleointensity and oxygen isotope data for the last 1.5 Myr (PISO-1500). *Earth and Planetary Science Letters* **283**, 14-23 (2009).

36 Lund, S., Stoner, J. S., Channell, J. E. & Acton, G. A summary of Brunhes paleomagnetic field variability recorded in Ocean Drilling Program cores. *Physics of the Earth and Planetary Interiors* **156**, 194-204 (2006).

37 Channell, J. Late brunhes polarity excursions (mono lake, laschamp, iceland basin and pringle falls) recorded at odp site 919 (Irminger basin). *Earth and Planetary Science Letters* **244**, 378-393 (2006).

38 Channell, J., Riveiros, N. V., Gottschalk, J., Waelbroeck, C. & Skinner, L. Age and duration of Laschamp and Iceland Basin geomagnetic excursions in the South Atlantic Ocean. *Quaternary science reviews* **167**, 1-13 (2017).

39 Laj, C., Guillou, H. & Kissel, C. Dynamics of the Earth magnetic field in the 10–75 kyr period comprising the Laschamp and Mono Lake excursions: New results from the French Chaîne des Puys in a global perspective. *Earth and Planetary Science Letters* **387**, 184-197 (2014).

40 Singer, B. S., Guillou, H., Jicha, B. R., Zanella, E. & Camps, P. Refining the Quaternary geomagnetic instability time scale (GITS): Lava flow recordings of the Blake and Post-Blake excursions. *Quaternary Geochronology* **21**, 16-28 (2014).

41 Channell, J. The Iceland Basin excursion: Age, duration, and excursion field geometry. *Geochemistry, Geophysics, Geosystems* **15**, 4920-4935 (2014).

42 Laj, C. & Channell, J. Geomagnetic excursions, *in Treatise on Geophysics*. *Geomagnetism* **5**, 373-416 (2007).

43 Roberts, A. P., Tauxe, L. & Heslop, D. Magnetic paleointensity stratigraphy and high-resolution Quaternary geochronology: successes and future challenges. *Quaternary Science Reviews* **61**, 1-16 (2013).
